# SFRP4^+^ stromal cell subpopulation with IGF1 signaling in human endometrial regeneration

**DOI:** 10.1038/s41421-022-00438-7

**Published:** 2022-09-27

**Authors:** Bingbing Wu, Yu Li, Nanfang Nie, Xilin Shen, Wei Jiang, Yanshan Liu, Lin Gong, Chengrui An, Kun Zhao, Xudong Yao, Chunhui Yuan, Jinghui Hu, Wei Zhao, Jianhua Qian, XiaoHui Zou

**Affiliations:** 1 Clinical Research Center, the First Affiliated Hospital, School of Medicine, Zhejiang University, Hangzhou, Zhejiang China; 2 Dr. Li Dak Sum & Yip Yio Chin Center for Stem Cell and Regeneration Medicine, Zhejiang University, Hangzhou, Zhejiang China; 3 Zhejiang Provincial Key Laboratory of Tissue Engineering and Regenerative Medicine, Hangzhou, Zhejiang China; 4 International Institutes of Medicine, The 4th Affiliated Hospital of Zhejiang University School of Medicine, Hangzhou, Zhejiang China; 5 Chu Kochen Honors College, Zhejiang University, Hangzhou, Zhejiang China; 6 Zhejiang University-University of Edinburgh Institute, Hangzhou, Zhejiang China; 7 Department of Gynecology, the First Affiliated Hospital, School of Medicine, Zhejiang University, Hangzhou, Zhejiang China

**Keywords:** Cell division, Regeneration

## Abstract

Our understanding of full-thickness endometrial regeneration after injury is limited by an incomplete molecular characterization of the cell populations responsible for the organ functions. To help fill this knowledge gap, we characterized 10,551 cells of full-thickness normal human uterine from two menstrual phases (proliferative and secretory phase) using unbiased single cell RNA-sequencing. We dissected cell heterogeneity of main cell types (epithelial, stromal, endothelial, and immune cells) of the full thickness uterine tissues, cell population architectures of human uterus cells across the menstrual cycle. We identified an SFRP4^+^ stromal cell subpopulation that was highly enriched in the regenerative stage of the human endometria during the menstrual cycle, and the SFRP4^+^ stromal cells could significantly enhance the proliferation of human endometrial epithelial organoid in vitro, and promote the regeneration of endometrial epithelial glands and full-thickness endometrial injury through IGF1 signaling pathway in vivo. Our cell atlas of full-thickness uterine tissues revealed the cellular heterogeneities, cell population architectures, and their cell–cell communications during the monthly regeneration of the human endometria, which provide insight into the biology of human endometrial regeneration and the development of regenerative medicine treatments against endometrial damage and intrauterine adhesion.

## Introduction

The human uterine organ, especially the full-thickness endometria, is essential for fertilization and embryonic development. Human endometria, which mainly comprise endometrial epithelial and stromal cells, exhibit remarkable plasticity and undergo repeated injury and regeneration^1,2^. The highly dynamic properties of repeated injury and scar-less repair during the menstrual cycle make it an ideal model to study tissue regeneration^3^. Full-thickness injury or dysfunction of the human endometria causes intrauterine adhesion, miscarriage, and uterine factor infertility. The development of new regenerative technologies against intrauterine adhesion, miscarriage, and infertility diseases is hindered by our incomplete understanding of the molecular characterization of the cell populations responsible for scar-less endometrial regeneration during the menstrual cycle.

The tissue microenvironment is indispensable during tissue development^4^, homeostasis^5^, regeneration, and disease progression^6^. Single cell analysis has been increasingly utilized to dissect cell heterogeneity and study dynamic cell population architectures and their regulation during biological processes such as development, tissue homeostasis, and pathology^4–6^. Organoid technology has been increasingly utilized to study cell-cell interactions within the tissue microenvironment^7,8^. Thus, in this study, we dissected cell heterogeneity of main cell types of full-thickness uterine tissues, identified an SFRP4^+^ stromal cell subpopulation that are enriched in the regenerative stage of the endometria during the menstrual cycle as potential regenerative cell populations, and determined that the SFRP4^+^ stromal cells could significantly enhance the proliferation of human endometrial epithelial organoid in vitro. In addition, this study found that promotion of the regeneration of endometrial epithelial glands and full thickness endometrial injury occurred through the insulin-like growth factor 1 (IGF1) signaling pathway in vivo. Our cell atlas of full-thickness uterine tissues revealed cellular heterogeneities, cell population architectures, and their communication during the monthly regeneration of human endometria, which provides insight into the biology of human endometrial regeneration and the development of regenerative medicine treatments against endometrial damage and intrauterine adhesion.

## Results

### Single cell RNA-seq profiling and unbiased clustering of cells from full thickness human uterine tissues

**Fig. 1:**
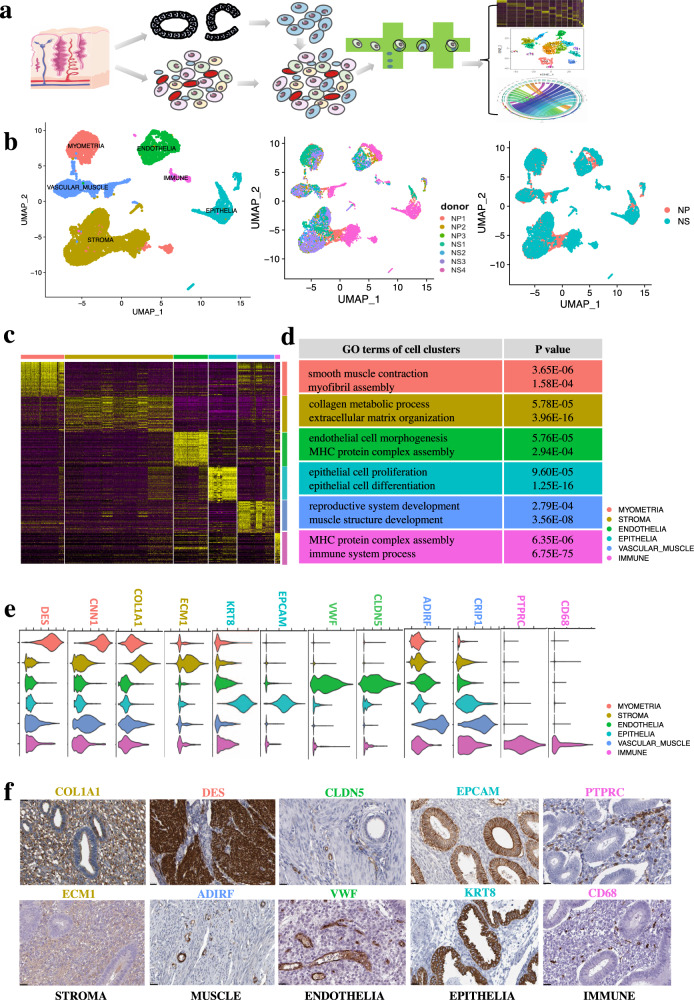
Single cell RNA-seq profiling and unbiased clustering of cells from full thickness human uterine tissues. **a** Workflow shows sample processing, enzymatic digestion and drop-seq based single cell RNA-seq. **b** UMAP plot of 6 main clusters, donor ID and secretory/proliferative origin phases of full-thickness uterus by single cell RNA-seq (scRNA-seq). **c** Heatmap shows specifically expressed gene signature of the 6 main clusters of full-thickness uterus. **d** Gene ontology (GO) analysis of the specifically expressed gene signature of the 6 main clusters of full-thickness uterus. **e** Violin plot showed specific marker genes from gene signatures of each cell cluster (DES, CNN1 for myometrial smooth muscle cells; COL1A1, ECM1 for stroma cells; KRT8, EPCAM for epithelial cell; CLDN5, VWF for endothelial cells; ADIRF, CRIP1 for vascular smooth muscle cells; PTPRC, CD68 for immune cells). **f** Immunohistochemistry staining from the HPA further validated the expression of specific markers of the 6 main clusters: COL1A1 (https://www.proteinatlas.org/ENSG00000108821-COL1A1/tissue/endometrium#img), ECM1 (https://www.proteinatlas.org/ENSG00000143369-ECM1/tissue/endometrium#img) was expressed in the uterus stromal cells, DES (https://www.proteinatlas.org/ENSG00000175084-DES/tissue/endometrium#img) was expressed in the uterine myometrial smooth muscle cells, ADIRF (https://www.proteinatlas.org/ENSG00000148671-ADIRF/tissue/endometrium#img) was expressed in the uterine vascular smooth muscle cells, CLDN5 (https://www.proteinatlas.org/ENSG00000184113-CLDN5/tissue/endometrium#img), VWF (https://www.proteinatlas.org/ENSG00000110799-VWF/tissue/endometrium#img) were expressed in the uterus endothelial cells, EPCAM (https://www.proteinatlas.org/ENSG00000119888-EPCAM/tissue/endometrium#img), KRT8 (https://www.proteinatlas.org/ENSG00000170421-KRT8/tissue/endometrium#img) were expressed in the uterus epithelial cells,, PTPRC (https://www.proteinatlas.org/ENSG00000081237-PTPRC/tissue/endometrium#img), CD68 (https://www.proteinatlas.org/ENSG00000129226-CD68/tissue/endometrium#img) were expressed in the uterus immune cells. Scale bar, 25 µm.

First, we used droplet-based single-cell RNA-seq (10× Genomics Chromium system) to profile single-cell suspensions from seven full-thickness normal human uterine tissues from two menstrual phases (Supplementary Table S1) (3 from the proliferative-NP and 4 from secretory phase-NS) (Fig. 1a). We then used the Cell Ranger Pipeline (10× Genomics) to map the raw sequencing data. We isolated and profiled 10,942 individual cells from human uterine tissue. We then filtered the data based on the number of counts (nCount_RNA < 60,000), features (nFeature_RNA > 500), and mitochondrial counts (percent. mt < 10) of each cell (Supplementary Fig. S1a–d), after computational quality control 10,551 individual cells were left, and the FindIntegrationAnchors function from the Seurat package^9^ was used to integrate the transcriptomes of the filtered cells from the two groups (NP & NS). We then selected the highly variable feature genes from 2000 feature genes using the FindVariableFeatures function from the Seurat package^9^ as visualized in the Elbow plot (Supplementary Fig. S1e). According to the variance of each principal component (PC), we selected genes in PC 1–16 to perform the downstream graph-based clustering of the filtered cells and partitioned all the cells into six main clusters, donor ID and secretory/proliferative origin phases of full-thickness uterus, which were visualized using Uniform Manifold Approximation and Projection (UMAP) (Fig. 1b). The potential doublet of the single-cell data was detected using DoubleFinder^10^ (Supplementary Fig. S1f); each cluster possesses a unique set of marker genes (Fig. 1c) and gene ontology (Fig. 1d; Supplementary Tables S2, S3). As labeled by specifically expressed marker genes and gene ontologies, we named the main clusters as endometrial epithelia, stroma, endothelia, smooth muscle, and immune cells in the human uterus.

Myometrial smooth muscle showed elevated levels of *DES* and *CNN1* (Fig. 1e). Specific genes expressed in myometrial smooth muscle cells were enriched in Gene ontology (GO) terms of regulation of smooth muscle contraction and myofibril assembly (Fig. 1d; Supplementary Table S3). Endometrial stromal cells expressed elevated levels of *COL1A1* and *ECM1* (Fig. 1e). Specific genes expressed in stromal cell populations were enriched in GO terms of collagen metabolic process and extracellular structure organization (Fig. 1d; Supplementary Table S3). Endothelial cells expressed elevated levels of *VWF* and *CLDN5* (Fig. 1e). Specific genes expressed in endothelial cells showed enriched GO terms of endothelial cell morphogenesis and MHC protein complex assembly (Fig. 1d; Supplementary Table S3). Endometrial epithelial cells expressed high levels of *KRT8* and *EPCAM* (Fig. 1e). Specific genes expressed in epithelial populations showed enriched GO terms of epithelial cell proliferation and epithelial cell differentiation (Fig. 1d; Supplementary Table S3). Vascular smooth muscle showed high levels of *ADIRF* and *CRIP1* (Fig. 1e). Specific genes expressed in muscle cells showed enriched GO terms of reproductive system development and muscle structure development (Fig. 1d; Supplementary Table S3). Endometrial immune cells expressed high levels of *PTPRC* and *CD68* (Fig. 1e). Specific genes expressed in immune cell populations showed enriched GO terms of MHC protein complex assembly and immune system processes (Fig. 1d; Supplementary Table S3). Immunohistochemistry (IHC) images from the Human Protein Atlas (HPA) (http://www.proteinatlas.org/)^11^ further validated the expression of specific markers of the five main cell populations as follows: ECM1 and COL1A1 were expressed in the uterine stromal cells, DES was expressed in the uterine myometrial cells, ADIRF was expressed in the uterine vascular muscle cells, VWF and CLDN5 were expressed in the uterine endothelial cells, EPCAM and KRT8 were expressed in the uterine epithelial cells, and PTPRC and CD68 were expressed in the uterine immune cells (Fig. 1f).

Each main cluster can be further clustered into subpopulations. In this study, we found 20 distinct subpopulations in total from six main groups of full-thickness uterine tissues using single-cell technology. The uterine epithelial cells could be further clustered into five subpopulations, each of which possesses a unique set of genes and gene ontology (Supplementary Fig. S2a, Tables S4, S5). We named the epithelial subpopulations as antigen-presenting epithelia, EMT epithelial, secretory epithelia, proliferative epithelial, and ciliated epithelia (Supplementary Fig. S2a). Uterine endothelial cells could be clustered into two subpopulations, including inflammatory endothelial and secretory endothelial cells (Supplementary Fig. S2b, Tables S8, S9). The uterine vascular smooth muscle cells were clustered into four subpopulations: ADIRF^+^ vascular, secretory vascular, inflammatory vascular, and DES^+^ vascular smooth muscle cells (Supplementary Fig. S2c, Tables S10, S11). The immune cells of the human uterus clustered into macrophages and NK cells (Supplementary Fig. S2d, Tables S12, S13). Uterine myometrial cells were further clustered into three subpopulations: MFAP5^+^ myometrial, DCN^+^ myometrial, and secretory myometrial muscle cells (Supplementary Fig. S2e, Tables S14, S15).

### Cell population architectures across the menstrual cycle identified SFRP4^+^ stroma cells as potential regenerative endometrial cell populations

**Fig. 2:**
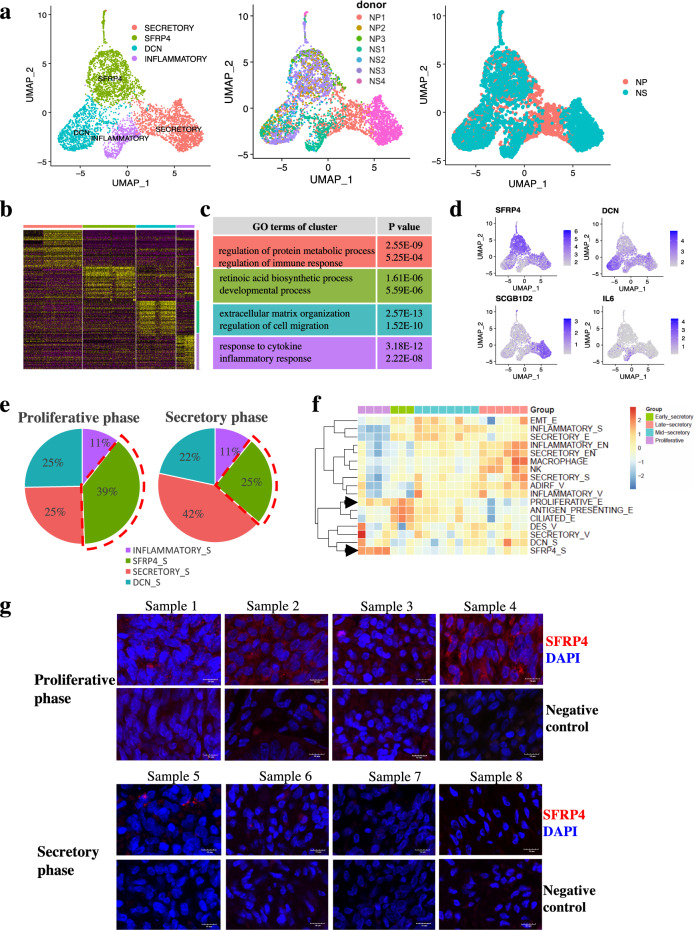
Cell population architectures across the menstrual cycle identified SFRP4^+^ stroma cells as potential regenerative endometrial cell populations. **a** UMAP plot of four uterus stroma cell sub-populations, donor ID and secretory/proliferative origin phases using Seurat. **b** Heatmap shows differential expressed gene signature of each sub-cluster from stromal cells. **c** Gene ontology (GO) analysis of the specifically expressed gene signature of each sub-population from stroma cells. **d** Featureplot depicted specific markers for each stroma sub-population (*SCGB1D2* for secretory stroma cells; *SFRP4* for SFRP4^+^ stroma cells; *DCN* for DCN^+^ stroma cells; *IL6* for inflammatory stroma cells). **e** Cell proportions of endometrial stromal in the proliferative and secretory phase of the endometria. **f** Heatmap showed relative proportional score of each subpopulation in endometria during the menstrual cycle from proliferative, early-secretory, mid-secretory to late-secretory phase of human endometria, the black arrow highlighted the proliferative epithelia and SFRP4^+^ stroma with high relative proportional score in proliferative phase. **g** Immunofluorescence staining of SFRP4^+^ stroma in different phase of menstrual cycle. Scale bar, 10 µm.

Uterine endometrial stromal cells clustered into four subpopulations (Fig. 2a). As labeled by specifically expressed marker genes and gene ontologies (Fig. 2b, c; Supplementary Tables S6, S7), we named the uterus stromal subpopulations as secretory stroma, SFRP4^+^ stroma, DCN^+^ stroma, and inflammatory stroma. As shown by the feature plot, secretory stroma expressed elevated levels of *SCGB1D2*, SFRP4^+^ stromal cells expressed high levels of *SFRP4*, DCN^+^ stroma expressed elevated levels of *DCN*, and inflammatory stroma expressed high levels of *IL6* (Fig. 2d). Specific genes expressed in stromal subpopulations enriched GO terms of regulation of protein metabolic process and regulation of immune response in secretory stroma, retinoic acid biosynthetic process, and developmental process in SFRP4^+^ stroma, extracellular matrix organization, and regulation of cell migration in DCN^+^ stroma, and response to cytokine and inflammatory response in inflammatory stroma (Fig. 2c).

The uterine endometria would undergo regeneration and differentiation under the influence of hormones during the menstrual cycle. Although there have been some relevant studies based on bulk tissues^12^, some recent single-cell studies on human endometria have failed to reveal the underlying mechanisms of regeneration and differentiation of human endometria at the single-cell level. Therefore, we then investigated the effect of the menstrual cycle on the cellular and molecular dynamics of the endometrial cell population architecture and further revealed their regeneration and differentiation hierarchies.

To reconstruct the temporal dynamics of all cell populations during the menstrual cycle, we first calculated the relative proportion of all cell populations by deconvolution analysis, using marker genes of each cell population generated in our cell atlas on a published dataset (GSE4888) on transcriptional profiling of bulk human endometrium^12^ (Supplementary Fig. S3). There were mainly four patterns of endometrial cell population architectures from proliferative through the early, mid-secretory to late secretory phase of the menstrual cycle (Fig. 2f) and are as follows: Pattern 1, the SFRP4^+^ stromal cell populations dominated the first pattern; the proportion increased mainly in the proliferative phase and decreased in the other phases of the endometria; Pattern 2, the proliferative epithelial, ciliated epithelial, and antigen-presenting epithelial populations dominated the second pattern that increased from the proliferative phase to the early secretory phase and decreased afterwards; Pattern 3, inflammatory stroma, EMT epithelial and secretory epithelial cell populations of the third pattern dominated in the mid-secretory phase of the menstrual cycle; Pattern 4, the rest of the cell populations mainly dominated the fourth pattern that increased only in the late secretory phase of the menstrual cycle, which was consistent with previous results that NK cell subsets were reported to be abundant in the late secretory phase of the endometria that rebuild and maintain appropriate local microenvironment for pregnancy^13^. Monocytes/macrophages are responsible for the breakdown and are associated with repair and remodeling^14^.

As the dynamics of the cell population architectures shown above were highly correlated with the menstrual cycle, we investigated the potential cell populations responsible for endometrial regeneration. The SFRP4^+^ endometrial stromal cell population was shown to dominate the first pattern, mainly in the proliferative phase, and we validated the results by immunofluorescence staining of SFRP4^+^ stroma in different phases of the menstrual cycle. SFRP4^+^ stromal cells were highly enriched in samples from the proliferative phase of the endometria compared with those from the secretory phase of the endometria (Fig. 2e, g). Thus, our results are consistent with the deconvolution analysis that identified SFRP4^+^ stromal cells as potential regenerative endometrial cell populations. To further confirm the above results, we used external single-cell data^15^ to verify SFRP4^+^ stroma as proliferative phase-specific endometrial cell populations, which depicted specific markers of SFRP4 for SFRP4^+^ stromal cells in the external single cell data (Supplementary Fig. S4a, b). Different proportions of stromal subsets in distinct phases of the endometrium during the menstrual cycle showed that SFRP4^+^ stromal cells were specifically highly enriched in the proliferative phase of the endometria, accounting for more than 90% of the stromal cells from the proliferative phase of the endometria (Supplementary Fig. S4c). As SFRP4^+^ stroma was specifically enriched in proliferative endometria, we inferred that SFRP4^+^ stroma is a potential regenerative endometrial cell population.

Next, we examined the spatial distribution of SFRP4^+^ stromal cells. IHC staining images from the HPA showed that the SFRP4^+^ staining stromal cells were equally distributed in both the functional endometrial layer (Supplementary Fig. S5a), close to the uterine cavity, and the basal endometrial layer (Supplementary Fig. S5b), close to the myometrium, which was also consistent with the results that SFRP4^+^ stromal cells were in the proliferative phase of the endometrium. In addition, more than 90% of the stromal cells from the proliferative phase of the endometria were SFRP4^+^ stromal cells.

### Connectivity analysis revealing signals from SFRP4^+^ stroma cells promote endometrial epithelial organoid proliferation

**Fig. 3:**
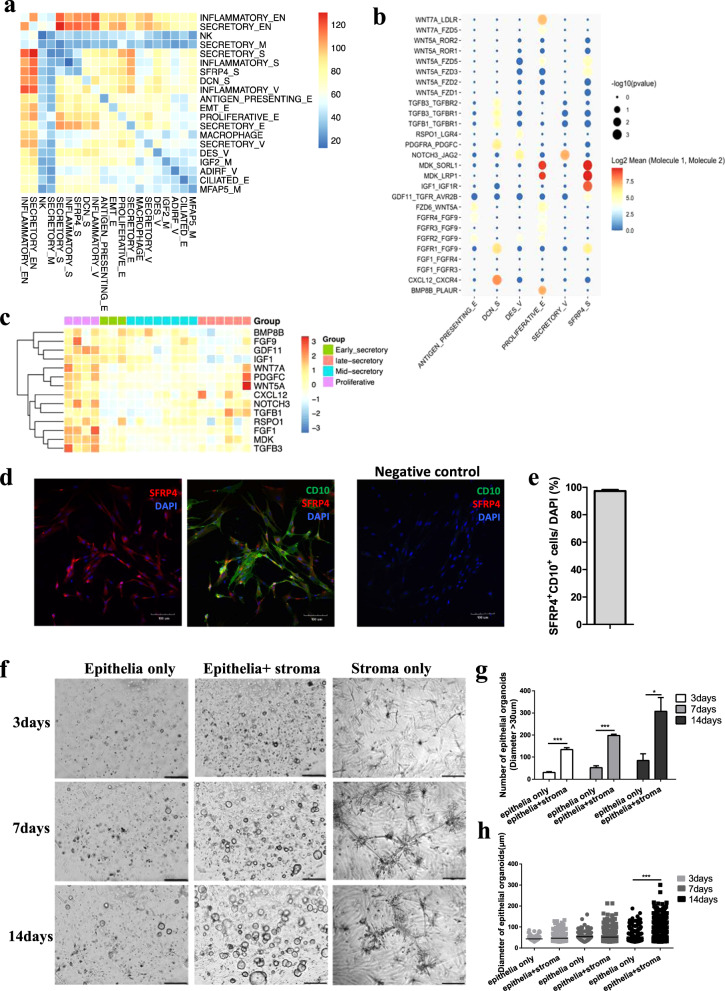
Connectivity analysis revealing signals from SFRP4^+^ stroma cells promote endometrial epithelial organoid proliferation in vitro. **a** Heatmap shows the total numbers of receptor-ligand interactions from any of the two sub-populations from the microenvironment of full-thickness human uterus in proliferative phase. **b** Clustering of the interactions of ligands (uniquely expressed in proliferative endometria) from each cell sub-population of the proliferative endometria to receptors from the proliferative epithelial cell sub-population. **c** Heatmap shows expression of ligands uniquely expressed in proliferative phase of the human endometria derived from another transcriptional dataset of the human endometria during the menstrual cycle (GSE4888). **d** immunofluorescence staining of co-expression with SFRP4 and CD10 in cultured human endometrial stromal cells, scale bar, 100 μm. **e** Percentage of SFRP4 and CD10 double positive cells in each high-power field. *n* = 6. **f** organoids culturing of endometrial epithelia cells with or without SFRP4^+^ stroma co-cultured, scale bar, 200 μm. **g** quantification of endometrial epithelial organoids at different time points of co-cuture, *p < 0.05; ***p < 0.001. **h** diameter of endometrial epithelial organoids at different time points of co-cuture, ***p < 0.001.

The tissue microenvironment is indispensable for tissue homeostasis and regeneration^5^. Different cell populations in tissues are surrounded by each other, and communication among cells regulates and balances cell populations to achieve proper regeneration^4,5^. Thus, we reconstructed the intrauterine connectivity map among the cell populations using CellPhoneDB^16^. Finally, we obtained 400 significant connections of 463 and 485 ligand—receptor pairs among 20 cell subpopulations from both the proliferative and secretory phases of the human uterus, respectively (Fig. 3a; Supplementary Fig. S6a–c, Tables S16, S17). As shown in the heatmaps, there were dramatic differences in terms of the total number of receptor-ligand interactions from any of the two subpopulations from the microenvironment of the full-thickness proliferative (Fig. 3a) and secretory (Supplementary Fig. S6a) human uterus.

Proliferation is the main stage during the regeneration process in the proliferative phase of endometria^17^. Thus, we analyzed the potential regulatory cellular microenvironment of proliferative epithelia using the connectivity map. According to the unique temporal distribution of proliferative epithelial and SFRP4^+^ stromal cell populations (Fig. 2f). We selected cell populations that showed a similar temporal distribution to that of the proliferative epithelia of human endometria as the microenvironment of the proliferative epithelia in unique. The connections of ligands from each cell population of the regenerative microenvironment to receptors from the proliferative epithelia showed that ligands (WNTs, FGFs, IGF1, and MDK) from the SFRP4^+^ stromal cell population regulated the proliferative epithelia in the proliferative phase of human endometria (Fig. 3b). We then validated the temporal expression patterns of the ligands surrounding the proliferative epithelia from the predicted connectivity map using a published dataset (GSE4888) for transcriptional profiling of the bulk human endometrium^12^. We selected ligands with unique expression patterns that were highly correlated with the temporal dynamics of the proliferative epithelial cell population (Fig. 2f). As shown in the heatmaps, similar to the pattern of the proliferative epithelia, ligands of *WNTs*, *FGFs*, *IGF1*, and *GDFs* superfamily members were highly expressed in the proliferative phase of human endometria (Fig. 3c).

To further validate the proliferative effect of the SFRP4^+^ endometrial stromal cell population on endometrial epithelia, we first cultured stromal cells from endometria in the proliferative phase of the menstrual cycle. As shown in the immunofluorescence staining with SFRP4 and CD10 (Fig. 3d), approximately 97.28% of the cultured endometrial stromal cells were SFRP4 and CD10 double-positive cells (Fig. 3e), which was consistent with the single-cell data showing that most of the stromal cells in the proliferative phase of the endometria were SFRP4^+^ endometrial stromal cells (Supplementary Figs. S4c, S5). Next, we co-cultured cultured SFRP4^+^ endometrial stromal cells with in vitro endometrial epithelial organoids and found that SFRP4^+^ endometrial stromal cells promoted the proliferation of endometrial epithelial organoids after 3, 7, and 14 days of co-culture (Fig. 3f). Quantification of endometrial epithelial organoids at different time points of co-culture showed that SFRP4^+^ endometrial stromal cells could significantly increase the number and diameter of endometrial epithelial organoids compared with those cultured alone (Fig. 3g, h).

### SFRP4^+^ stromal cells promote the proliferation of endometrial epithelial organoid in vitro through regenerative IGF1 signaling

To study the underlying mechanisms by which SFRP4^+^ stromal cells promote the proliferation of endometrial epithelial organoids, we aimed to identify the key signaling molecules and pathways. The result showed that the SFRP4^+^ stromal cell population was shown to promote the proliferation of endometrial epithelia, most likely through the secretion of ligands (Fig. 3b).

**Fig. 4:**
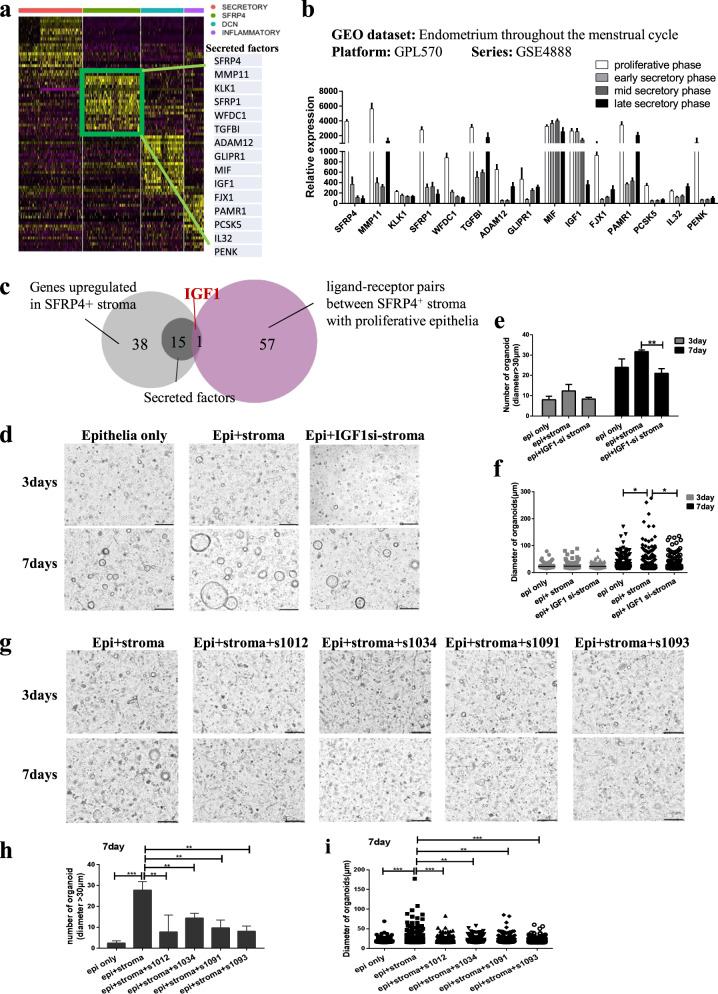
SFRP4^+^ stromal cells promote the proliferation of endometrial epithelial organoid in vitro through regenerative IGF1 signaling. **a** Heatmap showed differentially expressed genes between SFRP4^+^ stromal cells and other stromal cell subsets. Among them, 15 secreted factors were significantly highly expressed in SFRP4^+^ stroma. **b** the transcriptional dataset (series: GSE4888) verified that the 15 secretory factors were specifically highly expressed in proliferative phase. **c** Venn diagram of genes significantly highly expressed in SFRP4^+^ stroma (light gray) compared to secretory factors (dark gray), and interaction clusters of ligands from SFRP4^+^ stroma to receptors from the proliferative epithelial subset (purple). **d** organoids culturing of endometrial epithelia cells cocultured with SFRP4^+^ stroma from normal or IGF1 knockdown (IGF1si-stroma) cells, scale bar, 200 μm. **e** quantification of endometrial epithelial organoids at different time points of co-cuture, **p < 0.01; **f** diameter of endometrial epithelial organoids at different time points of co-cuture, *p < 0.05. **g** organoids culturing of endometrial epithelia cells with or without IGF1 inhibitors when cocultured with SFRP4^+^ stroma, 4 IGF1 inhibitors (s1012, s1034, s1091, s1093) are from Selleck.cn, scale bar, 200 μm. **h** quantification of endometrial epithelial organoids in different groups of co-culture, **p < 0.01; ***p < 0.001. **i** diameter of endometrial epithelial organoids in different groups of co-cuture, **p < 0.01; ***p < 0.001.

Firstly, we showed differentially expressed genes between SFRP4^+^ stromal cells and other stromal cell subsets, among which 15 secreted ligands were significantly highly expressed in SFRP4^+^ stromal cells (Fig. 4a). We further verified that the 15 secreted ligands were specifically highly expressed in the proliferative phase in a public dataset (GEO series: GSE4888)^12^ (Fig. 4b). The Venn diagram showed that IGF1 was the only overlap (Fig. 4c) between the 15 secreted ligands (dark gray) significantly highly expressed in SFRP4^+^ stroma (light gray) and the interaction of ligands from SFRP4^+^ stroma with receptors from the proliferative epithelial subset (purple) (Fig. 3b). Thus, IGF1 is potentially the key regenerative signaling molecule secreted by SFRP4^+^ endometrial stromal cells that promotes the proliferation of endometrial epithelia.

Next, we studied the effect of IGF1 on endometrial epithelia. The 3D organoid culture showed that IGF1 supplementation could also promote organoid formation (Supplementary Fig. S7a), with a significant increase in the number (Supplementary Fig. S7b) and diameter (Supplementary Fig. S7c) of endometrial epithelial organoids compared to those without IGF1 supplementation, which was consistent with the effect of the SFRP4^+^ stroma on endometrial epithelial organoids (Fig. 3f). We also found that IGF1 supplementation promoted the migration of endometrial epithelia in the 2D endometrial epithelial culture system (Supplementary Fig. S7d–f).

Next, we investigated whether SFRP4^+^ stroma promotes endometrial epithelial organoid formation through IGF1 signaling. We used siRNA to knock down *IGF1* expression in the SFRP4^+^ stromal cells, three different siRNAs were designed according to different regions of the human *IGF1* mRNA sequence (IGF1-si-1, IGF1-si-2, IGF1-si-3). qPCR was conducted to validate the efficiency of each siRNA 24 h after each of the three siRNAs against *IGF1* were transfected into the SFRP4^+^ stromal cells. The results showed that both IGF1-si-1 and IGF1-si-3 could significantly knock down the expression of *IGF1* in the SFRP4^+^ stromal cells, and IGF1-si-3 showed the best performance, therefore we chose IGF1-si-3 for the rest of the experiments (Supplementary Fig. S8). After IGF1-si-3 was transfected into SFRP4^+^ stromal cells, the stromal cells were then co-cultured with endometrial epithelial organoids. The results showed that IGF1 knockdown (IGF1si-stroma) in SFRP4^+^ stromal cells significantly hindered the formation of epithelial organoids (Fig. 4d), with a significant decrease in the number (Fig. 4e) and diameter (Fig. 4f) of organoids formed compared to those that were co-cultured with normal SFRP4^+^ stromal cells. We also confirmed that SFRP4^+^ stroma promotes endometrial epithelial organoid formation through IGF1 signaling using four IGF1 signaling pathway inhibitors (s1012, s1034, s1091, and s1093). The results showed that supplementation of any of the four inhibitors into the organoid culture system co-cultured with SFRP4^+^ stromal cells abolished the enhancement effects of SFRP4^+^ stromal cells on endometrial epithelial organoids (Fig. 4g), with a significant decrease in the number (Fig. 4h) and diameter (Fig. 4i) of organoids formed compared to those cultured without the IGF1 signaling pathway inhibitor. In this section, we identified the key IGF1 signaling molecules secreted by SFRP4^+^ stromal cells that promote the proliferation of endometrial epithelial organoids in vitro.

We also conducted the effects of IGF1 and knockdown or inhibition of IGF1 on stromal cell proliferation (Supplementary Fig. S8b, c). As the results showed that knockdown of *IGF1* mRNA using IGF1 siRNA did not affect cell proliferation of stroma cells (Supplementary Fig. S8b), while complete inhibition of IGF1 signaling using receptor inhibitors of IGF1 signaling could inhibit the cell proliferation of stroma cells compared with the control (Supplementary Fig. S8c). The probable explanation is as follows: though knockdown of *IGF1* mRNA using IGF1 siRNA significantly decrease the expression of IGF1, IGF1 siRNA could not completely shut-down the expression of IGF1, there might be still basal constitutive expression of IGF1 even after knockdown of *IGF1* mRNA, while inhibition of IGF1 signaling using receptor inhibitors of IGF1 signaling could completely inhibit the IGF1 signaling, which explained the decrease in cell proliferation of stromal cells.

### SFRP4^+^ stromal cells promote the regeneration of endometrial epithelial glands and full thickness endometrial injury through IGF1 signaling pathway in vivo

Next, we studied the regenerative potential of SFRP4^+^ endometrial stromal cells in an in vivo full-thickness endometrial injury model along with the effect of the key IGF1 signal molecule secreted from SFRP4^+^ stromal cells. A rat model of full-thickness endometrial injury was constructed (Supplementary Fig. S9). SFRP4^+^ endometrial stromal cells were mixed with gelatin methacryloyl (GelMA) hydrogel^18^ and transplanted into the injury site of the rat full-thickness endometrial injury model.

The GelMA hydrogel was gelated under UV irradiation for 15 s (Supplementary Fig. S10a). Comparing the ^1^H-NMR spectra (Nuclear magnetic resonance spectrometer, AVANCE III, Switzerland) of gelatin and GelMA, new signals of the acrylic protons of methacrylic functions and methyl function can be observed at δ = 5.3 ppm, δ = 5.5 ppm and at δ = 1.8 ppm. Therefore, we concluded that methacrylate (MA) was successfully grafted onto gelatin (Supplementary Fig. S10b). The microstructures of the GelMA hydrogels were observed by scanning electron microscopy (SEM). The SEM images revealed uniform porous microstructures throughout all samples (Supplementary Fig. S10c). To detect the cytocompatibility of the GelMA hydrogel, we encapsulated and cultured fibroblast L929 in the hydrogel. According to the results of cell live/death staining, several dead cells (red color) were detected on the first day but disappeared on the seventh day (Supplementary Fig. S10d). The results showed that cell viability was 70% on the first day and 100% on the third and seventh days, indicating that the cells encapsulated in the hydrogel were active after long-term culture (Supplementary Fig. S10e). We also collected GelMA hydrogel extract at 24 h for cell culture. The results showed that there was no significant difference in the cell proliferation rate between cells cultured in hydrogel extract medium and normal medium (Supplementary Fig. S10f). These results indicate that the GelMA hydrogel had good cytocompatibility.

The IGF1 signaling pathway inhibitor (s1091) was previously proven safe after in vivo administration and already used in a phase 3 study^19^, therefore, s1091 was also mixed into the hydrogel together with SFRP4^+^ endometrial stromal cells to study the involvement of the key IGF1 signaling pathway during the regeneration of full-thickness endometrial injury after SFRP4^+^ stromal cell transplantation in vivo.

**Fig. 5:**
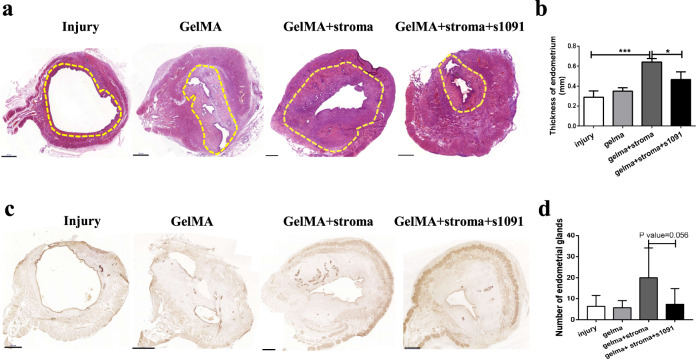
SFRP4^+^ stromal cells promote the regeneration of endometrial epithelial glands and full thickness endometrial injury through IGF1 signaling pathway in vivo. **a** H&E staining of rat uterus 1 week after operation in endometrial injury group (injury), material only group (Gelma), SFRP4^+^ stromal cell therapy group (Gelma + stroma) and cell compound IGF1 inhibitor group (Gelma + stroma + s1091). **b** comparison of endometrial thickness in different operation groups, *n* = 5, *p < 0.05; ***p < 0.001. **c** Immunohistochemical staining of FOXA2 showed endometrial glands 1 weeks after after operation in injury group, Gelma group, Gelma + stroma group and Gelma + stroma + s1091 group. **d** quantification of endometrial glands in in different operation groups, *n* = 5. scale bar, 500 μm.

The histology results showed that transplantation of the SFRP4^+^ stroma cells into the injury site promoted the regeneration of the rat endometria (Fig. 5a), with a thicker endometrial layer formed compared with that of the injury group (Fig. 5b). IHC staining of the endometrial epithelial glands using an anti-FOXA2 antibody showed that SFRP4^+^ stroma cells could promote new gland formation during the regeneration of the damaged endometria (Fig. 5c, d). The IGF1 signaling pathway inhibitor (s1091) abolished the regeneration of the SFRP4^+^ stroma cells both in full-thickness endometria and in the formation of new endometrial epithelial glands (Fig. 5b, d), which was consistent with the in vitro results that SFRP4^+^ stromal cells promote the formation of human endometrial epithelial organoids. In this section, we show that SFRP4^+^ stromal cells can promote the regeneration of endometrial epithelial glands and full-thickness endometrial injury through the IGF1 signaling pathway in vivo.

## Discussion and conclusion

Our understanding of full-thickness endometrial regeneration after injury is limited by the incomplete molecular characterization of the cell populations responsible for organ functions. Cell heterogeneity, cell population architecture, and regulation of complex tissues and organs are vital for tissue development, homeostasis, regeneration, and pathology^4–6^, however, the precise molecular mechanisms remained unknown until the broad applications of single-cell RNA-seq. In this study, we reconstructed the cell subpopulation architectures and communications map of full-thickness human uterine tissues from two menstrual phases (proliferative and secretory phases) using unbiased single-cell RNA-sequencing. Our study further sheds light on an SFRP4^+^ endometrial stromal cell subpopulation that is highly enriched in the regenerative stage of the endometria during the menstrual cycle. SFRP4^+^ stromal cells significantly enhanced the proliferation of endometrial epithelial organoids in vitro and promoted the regeneration of endometrial epithelial glands and full-thickness endometrial injury through the IGF1 signaling pathway in vivo. Our cell atlas of full-thickness uterine tissues revealed the cellular and molecular mechanisms regulating the monthly regeneration of human endometria, which provides insights into the biology of human endometrial regeneration and the development of regenerative medicine treatments against endometrial damage and intrauterine adhesion.

Most previous studies on uterine biology were based on bulk uterus/endometrium tissue transcriptomic analysis^20^ or a comparison between the different regions of the tissue^21^. With advances in technology and development analysis pipelines, studies have reached the single-cell level^22–25^. To the best of our knowledge, all cell populations throughout the menstrual cycle were included in our study, which provided the most detailed and dynamic cell populations of the uterine tissue to date in comparison with previous bulk, single-cell studies on endometrial tissue or in vitro endometrial organoids^15,24,26,27^.

Specifically, in comparison with two recent single cell study on endometria during the menstrual cycle^15,25^, our study provided more detailed and dynamic cell populations of the uterus tissue across the menstrual cycles, with a total of 20 functional distinct sub-populations identified which could be further grouped into 6 main clusters named as endometrial epithelia, stroma, endothelia, smooth muscle, and immune cells of the full-thickness uterus tissues by using the single-cell technology, and reconstructed the spatiotemporal cell population architectures of the full-thickness human uterus tissues during the menstrual cycle. In Wang et al. paper^15^, six cell types were identified: stromal fibroblast, endothelium, macrophage, lymphocyte, ciliated epithelium and unciliated epithelium, which was consistent with main clusters in our study. Their main findings failed to provide the subpopulation of the main endometrial cell, which did not fully exploit the advantages of the single-cell technology. Thus, the results from our study provided more detailed and heterogeneous cell populations of the uterus tissue across the menstrual cycles, and more abundant insight and resolution to the biology of the human endometria regeneration and differentiation.

And, in comparison with another single cell study on temporal and spatial dynamics of human endometria, we mainly fully characterized the stromal cell populations, while their study was mainly on the endometrial epithelial cells, with the focus on the molecular mechanisms on the differentiation of the epithelia towards secretory and ciliated lineages^25^, while our study was mainly focus on the regeneration of the endometria, which may provide complementary evidences to the whole picture of the human endometrial biology. In their study, 14 clusters of cells were identified, which could be grouped into five main cellular categories: (1) immune (lymphoid and myeloid); (2) epithelial (SOX9^+^, lumenal, glandular and ciliated); (3) endothelial (arterial and venous); (4) supporting—perivascular cells (PV STEAP4 and PV MYH11), smooth muscle cells and fibroblasts expressing C7 (fibroblasts C7); and (5) stromal–nondecidualized endometrial (eS) and decidualized endometrial (dS). The main differences between our clusters and clusters in Garcia-Alonso et al.'s paper^25^ were the epithelial and stromal cell clusters:

In our study, there were five epithelial clusters: Antigen_presenting, EMT, Secretory, proliferative, ciliated. Four epithelial clusters were reported in Garcia-Alonso et al’s paper^25^ (SOX9^+^, lumenal, glandular and ciliated) (1) SOX9 populations (MMP7^+^SOX9^+^); (2) ciliated cells (TPPP3^+^); (3) lumenal cells (PTGS1^+^PAX2^+^); and (4) glandular cells (SCGB2A2^+^). So, we map the expression of markers of the four epithelial clusters of theirs in our epithelial data (Supplementary Fig. S11a), and we find that marker of SOX9 populations (MMP7^+^SOX9^+^) is highly expressed in the EMT cluster in our study, which shows that the SOX9 populations in their paper is actually EMT cluster in our study, marker of ciliated cells (TPPP3^+^) is also highly expressed in CILIATED cluster in our study, which shows that the ciliated populations in their paper is the same as the CILIATED cluster in our study, marker of glandular cells (SCGB2A2^+^) is highly expressed in SECRETORY cluster in our study, which shows that the glandular populations in their paper is actually SECRETORY cluster in our study, and marker of lumenal cells (PTGS1^+^PAX2^+^) is highly expressed in ANTIGEN_PRESENTING cluster in our study, while the PROLIFERATIVE epithelial cluster in our study also express moderate level of the SOX9 populations marker MMP7. Thus, these results suggest that the epithelial clusters identified in our study were consistent and can be validated in the published single-cell datasets.

In case of stromal cell clusters, there were four epithelial clusters in our study: secretory stroma, SFRP4^+^ stroma, DCN^+^ stroma, and inflammatory stroma. As shown by the feature plot, secretory stroma expressed high levels of SCGB1D2, SFRP4^+^ stroma cells expressed high levels of SFRP4, DCN^+^ stroma expressed high levels of DCN, inflammatory stroma expressed high levels of IL6. There were three fibroblast/stromal clusters in Garcia-Alonso et al’s paper^25^: (1) Fibro C7 populations (C7^+^); (2) nondecidualized endometrial (eS) (MMP11^+^, CRABP2^+^) and (3) decidualized endometrial (dS) (CFD, IL15, FOXO1). So, we map the expression of markers of the three fibroblast/stromal clusters in their paper in our stromal cells data (Supplementary Fig. S11b), and we find that marker of C7 populations (C7) is highly expressed in the DCN cluster in our study, which shows that the C7 populations in their paper is actually DCN cluster in our study, marker of eS populations (MMP11, CARBP2) is highly expressed in the SFRP4 cluster in our study, which shows that the eS populations in their paper is actually SFRP4 cluster in our study, marker of dS populations (CFD, IL15, FOXO1) is highly expressed in the SECRETORY and INFLAMMATORY clusters in our study, which shows that the dS populations in their paper could be further divided into two clusters (SECRETORY and INFLAMMATORY) in our study. Thus, these results suggest that the stromal clusters identified in our study were also consistent and can be validated in the published single-cell datasets.

Previous studies have shown that endometrial stem/progenitor cells and endometrial mesenchymal stem cells are responsible for the regeneration of human and mouse endometria^2,28^. As there was an increasing attention on fibroblasts heterogeneity and functions in this field, fibroblasts were suspected to participate in tissue health and diseases through physical or biochemical niches by producing extracellular matrix or soluble signal molecules^29^. One of the populations being largely proposed as a regenerative population in the endometrium is the stromal SUSD2^+^, associated to perivascular areas^2,28^, so we checked the SUSD2^+^ cells in our data (Supplementary Fig. S11c, d), and found SUSD was highly enriched in the vascular cell population (ADIRF^+^ vascular cells) (Supplementary Fig. S11d), but there’s no positive expression in any of the stroma cells in our data (Supplementary Fig. S11c) and confirms the perivascular nature of the SUSD2^+^ cells, and also indicated that these cells were different from the regenerative SFRP4^+^ stroma cells found in our study. In this study, we revealed heterogeneity in endometrial stromal cells and identified a regenerative SFRP4^+^ endometrial stromal cell subpopulation that was specifically enriched in the regenerative stage of endometria during the menstrual cycle. SFRP4^+^ stromal cells significantly enhanced the proliferation of endometrial epithelial organoids in vitro and promoted regeneration of endometrial epithelial glands and full-thickness endometrial injury in vivo. These results highlight the crucial role of fibroblast-like stromal cells during menstrual endometrial repair and regeneration. SFRP4^+^ stromal cells also provide a novel cell source for tissue engineering and regenerative medicine treatment of endometrial injury, thin endometria, and intrauterine adhesions.

A previous study showed that IGF1 is involved in the regulation of reproductive tissues (such as endometria) and functions in the endometrium under the control of hormone receptors in mice^30^. In this study, we identified IGF1 as a key endogenous signaling molecule secreted by human SFRP4^+^ stromal cells during the regenerative phase of the menstrual cycle, which regenerates endometrial epithelial organoid formation in vitro, gland formation in vivo and full-thickness endometrial regeneration. This identification provided a promising bioactive molecule for tissue engineering and regenerative medicine treatment of endometrial injury, thin endometria, and intrauterine adhesion, as IGF1 was reported to be functional in the treatment of injury in peripheral Nerve^31^ and cartilage reconstruction in Osteoarthritis^32^. In addition to its role in the endometrial epithelia, IGF1 was also shown to promote interleukin 10 (IL10) expression in bone marrow stem cells, which suggests additional functions of IGF1 in immune regulation^33^.

## Materials and methods

### Human uterus collection

Seven full-thickness (including endometrium and myometrium layer) normal human uterine samples (all with normal menstrual cycle) from two menstrual phases (proliferative and secretory phase) were collected from the normal part of uterus from hysterectomy due to leiomyoma (Supplementary Table S1), after the whole uterus tissues were surgically removed from hysterectomy, the normal part of the uterus full-thickness (endometrium and myometrium layer) visible to the naked eye were selected to avoid the leiomyoma site. Tissues of about 1 cm in length, 1 cm in width and 2 cm in depth were immediately cut with a scalpel and transported to the laboratory in cell culture medium at a low temperature of 4 °C for subsequent tissue digestion experiments. All tissues were all collected from the First Affiliated Hospital, School of Medicine, Zhejiang University. Approval for utilizing the patient samples in this study was obtained from Ethics Committee of the First Affiliated Hospital, School of Medicine, Zhejiang University (Approval Reference Number: 2018-113). Patients taking any hormones were excluded from the study.

### Single cell suspension preparation

A single-cell suspension was prepared as described in a previous study^34^. Briefly, full-thickness uterus tissue was minced into small cubes with scissors, and digested in 20–30 mL digestion enzyme mixtures containing 1.25 U/mL Dispase II (Sigma, D4693)/0.4 mg/mL collagenase V (Sigma, C-9263) solution in RPMI 1640 medium (ThermoFisher Scientific, 21875-034) with gentle shaking at 37 °C for 20–30 min, with the digested tissue supernatant neutralized by 10% FBS in RPMI 1640 medium and replaced by new digestion enzyme mixtures every 20–30 min. Digested cells were collected, and red blood cells were removed using red cell lysis buffer (Beyotime Biotech, C3702). The stromal cells and smooth muscles in the neutralized digested tissue supernatant were collected by passing the digested supernatant through 70 μm cell sieves (Corning). The epithelial cells were backwashed and further digested with TrypLE (Thermo Fisher Scientific) at 37 °C for 10 min. The digested supernatant was passed through 70 μm cell sieves to obtain a single epithelial cell suspension. Finally, as we used the different digestion methods to get the stromal cells, myometrial muscles and epithelial cells separately, we need to combine the stromal cells, smooth muscles and epithelial cells to get the uterus single cell suspension for single cell analysis, the stromal cells, smooth muscles and epithelial cells were combined at a ratio of 1:1:1 to get the uterus single cell suspension for further single cell analysis (Fig. 1a).

### Single cell capture, pre-amplification and sequencing and Bioinformatic analysis

Single-cell capture and pre-amplification were conducted on a GemCode instrument (10× Genomics) according to the manufacturer’s ‘instructions (Chromium™ Single Cell 3’ Reagent Kit v2). The generated library was sequenced using the Illumina X10 platform and the generated sequencing reads were aligned and analyzed using the Cell Ranger Pipeline (10× Genomics). The raw count data of each single cell were deposited into the public database of the Genome Sequence Archive for Human (GSA-Human) under accession number HRA000928. Single-cell analysis was conducted using Seurat^9^. The potential doublet of single-cell data was detected using DoubleFinder^10^. A connectivity map was constructed according to a previous ligand-receptor dataset^35^ using CellPhoneDB^16^. GO analysis was conducted using http://geneontology.org. Gene set variation analysis (GSVA) was used to perform deconvolution analysis^36^.

### Human endometrial epithelial organoid co-cultured with SFRP4^+^ stroma

Single endometrial epithelial and stromal cell suspensions were digested, as described in the single-cell suspension preparation section. SFRP4 stromal cells were digested from human uterine samples during the proliferative phase. Cell suspensions were centrifuged and resuspended in ice-cold Matrigel (Corning, 536231) with 1 × 10^4^ epithelial cells and 0.5 × 10^4^ stroma per well. Drops of matrigel-cell suspension (20 μL) were plated into 48-well plates (Costar, 3548), allowed to set at 37 °C, and overlaid with 250 μL endometrial organoid expansion medium (ExM) with or without IGF1 inhibitors. Endometrial organoid expansion medium (ExM) was obtained from a previous study^34^, containing N2 supplement (Gibco), B27 supplement (Gibco), 50 ng/mL EGF (Peprotech), 100 ng/mL Noggin (Peprotech), 500 ng/mL R-spondin-1 (Peprotech), 100 ng/mL FGF10 (Peprotech), 50 ng/mL HGF (Peprotech), 500 nM ALK-4, -5, -7 inhibitor (Selleck, A83-01), 10 nM nicotinamide (Sigma), and 1.25 mM N-acetyl-L-cysteine (Sigma) in advanced DMEM/F12 medium (Gibco, C11330500BT). IGF1 inhibitors included BMS-536924 (Selleck, s1012) at a final concentration of 2 μM, NVP-AEW541 (Selleck, s1034) at a final concentration of 2.5 μM, linsitinib (Selleck, s1091) at a final concentration of 2 μM, GSK1904529A (Selleck, s1093) at a final concentration of 5 μM. The medium was changed every 2–3 days. All replicates for each one experiment were performed on organoids derived from a same individual biopsy, and we repeated the same experiment for three times, and we also repeated the organoid experiments using cells from different donors (marked in Supplementary Table S1). After endometrial epithelial organoid culture, the pictures were taken and saved. As shown in the figure below, the endometrial epithelial organoid spheres in the pictures are automatically recognized and selected with the count and measure object’s function plug-in in Image-Pro Plus 6.0 (the red logo is the endometrial epithelial organoid automatically recognized by the software), and the software calculates the number of epithelial organoids and the diameter of each epithelial organoid ball at the same time for further statistical analysis (Supplementary Fig. S8d).

### Transfection of siRNA

IGF1 siRNA were purchased from RiboBio. siRNA reagent was dissolved in a stock solution at a concentration of 20 μM. The transfection complex reagent, including 5 μL siRNA stock solution, 83 μL OPTI-MEM (Gibco), and 12 μL Lipo2000 (Invitrogen), was mixed and vortexed gently and incubated for 10 min at room temperature (20 °C). The transfection complex reagent was added to a 6-well plate with 2.5 × 10^5^ stromal cells and gently mixed with a final concentration of 50 nM siRNA in each well. The transfection reagent was replaced with fresh culture medium after 4 h incubation in 37 °C incubator. Stromal cells transfected with siRNAs were used for further analysis.

### Synthesis of GelMA

GelMA was fabricated as described previously^37^. Type A gelatin (Sigma-Aldrich) was dissolved in PBS) at 50 °C to obtain a 10% w/v homogeneous solution. Then a 0.1 mL methacrylic anhydride (MA) (Sigma-Aldrich) per gram of gelatin was added to the gelatin solution at a rate of 0.5 mL/min, with continuous stirring. The mixture was allowed to react at 50 °C for 3 h. The GelMA solution was dialyzed against deionized water using 8–14 kDa cutoff dialysis tubing (VWR Scientific USA) for 6 days at 50 °C to remove unreacted MA and any byproducts. The GelMA solution was frozen overnight at −80 °C, then lyophilized, and stored at −20 °C until further use. GelMA at a concentration of 10% was used to mix the cells in the in vivo study.

### Animal experiment

Natural-normal female SD rats (at the age of 8 weeks) were kept in a specific pathogen-free air-conditioned room and allowed free access to food and water at the Animal Center of Zhejiang University of Medicine. All experiments were approved by the Animal Experimental Ethical Inspection of the First Affiliated Hospital, College of Medicine, Zhejiang University (2018-095). Ten rats (including 20 uterine horns) were randomly divided into four groups (five uterine horns in each group): endometrial injury group (injury), material only group (GelMA), human SFRP4^+^ stromal cell therapy group (GelMA + stroma), and human SFRP4^+^ stromal cell compound IGF1 inhibitor group (GelMA + stroma + s1091). No immunosuppressive drugs were used during the animal experiments. Endometrial injury was induced as follows after the animal was anesthetized: a midline incision in the abdomen was made and the uterus was exposed. In the injury alone group, a 1 cm longitudinal incision was made on the opposite side of the mesometrium, with the endometrial layer exposed. The endometrial layers, with a length of 1 cm and 0.5 cm in width, were then torn off, and the smooth muscle layer remained intact (Supplementary Fig. S9). Finally, the injury sites were marked with 6-0 non-absorbable silk sutures, and the longitudinal incision wound was closed after endometrial injury. In the repair group, after injury, 15 μL GelMA hydrogel with or without 2 × 10^4^ stromal cells and s1091 were added to the injured wound site of each uterus, then irradiated by UV for 15 s to gelling in situ. The concentration of s1091 was 2 μM in the GelMA solution. After the surgery, the abdominal cavity was washed with 0.9% (w/v) normal saline. Then, the rectus abdominis, skin, and fascia were closed using sutures.

### Histology and immunofluorescence staining

Full-thickness normal human uterine tissues and rat uterine tissues were fixed in 4% (w/v) paraformaldehyde, dehydrated in an ethanol gradient, embedded in paraffin and sectioned at a 10 μm thickness. Then, the 10 μm-thick paraffin sections were stained with hematoxylin and eosin. Immunostaining was carried out as follows: The 10 μm paraffin sections were rehydrated, antigen retrieved, rinsed three times with PBS, and treated with blocking solution (1% BSA) for 1 h, prior to incubation with primary antibodies at 4 °C overnight. The primary antibodies SFRP4 (Novus, NBP2-76870), CD10 (Abcam, ab34199), and FOXA2 (IHCeasy, KHC0140) were used. Secondary antibodies, goat anti-rabbit Alexa Fluor 546 (Invitrogen, A11035), donkey anti-mouse Alexa Fluor 488 (Invitrogen, A21202), and DAPI (Beyotime, China), were used to visualize the respective primary antibodies and cell nuclei. All procedures were performed according to the manufacturer’s instructions.

### Statistical analysis

A quantitative comparison of the radius and number of organoids cultured in different conditioned media was conducted using ANOVA in PRISM 5.0, with all *P* values less than 0.05. considered statistically significant. Migration of endometrial epithelial cells in 2D culture media between groups of IGF1 supplement and the control was compared using an unpaired *t* test in PRISM 5.0, with all *P* values less than 0.05, considered statistically significant. Quantitative comparison of the thickness of regenerated endometria and number of glands formed among different groups was conducted using ANOVA in PRISM (version 5.0), with all *P* values less than 0.05, considered statistically significant. The expression of *IGF1* genes among groups transfected with different siRNAs against *IGF1* were compared using ANOVA in PRISM (version 5.0), with all *P* values less than 0.05, considered statistically significant.

## Supplementary information

Supplementary Figure S1-S11, Table S1

Supplementary Table S2

Supplementary Table S3

Supplementary Table S4

Supplementary Table S5

Supplementary Table S6

Supplementary Table S7

Supplementary Table S8

Supplementary Table S9

Supplementary Table S10

Supplementary Table S11

Supplementary Table S12

Supplementary Table S13

Supplementary Table S14

Supplementary Table S15

Supplementary Table S16

Supplementary Table S17

## References

[1] Ono M (2007). Side population in human uterine myometrium displays phenotypic and functional characteristics of myometrial stem cells. Proc. Natl. Acad. Sci. USA.

[2] Gargett CE, Nguyen HPT, Ye L (2012). Endometrial regeneration and endometrial stem/progenitor cells. Rev. Endocr. Metab. Dis..

[3] Maybin JA, Critchley HOD (2015). Menstrual physiology: implications for endometrial pathology and beyond. Hum. Reprod. Update.

[4] Camp JG (2017). Multilineage communication regulates human liver bud development from pluripotency. Nature.

[5] Zepp JA (2017). Distinct mesenchymal lineages and niches promote epithelial self-renewal and myofibrogenesis in the lung. Cell.

[6] Puram SV (2017). Single-cell transcriptomic analysis of primary and metastatic tumor ecosystems in head and neck cancer. Cell.

[7] Wan ACA (2016). Recapitulating cell-cell interactions for organoid construction - are biomaterials dispensable?. Trends Biotechnol..

[8] Bagley JA, Reumann D, Bian S, Levi-Strauss J, Knoblich JA (2017). Fused cerebral organoids model interactions between brain regions. Nat. Methods.

[9] Satija R, Farrell JA, Gennert D, Schier AF, Regev A (2015). Spatial reconstruction of single-cell gene expression data. Nat. Biotechnol..

[10] McGinnis CS, Murrow LM, Gartner ZJ (2019). DoubletFinder: doublet detection in single-cell RNA sequencing data using artificial nearest neighbors. Cell Syst..

[11] Uhlen M (2015). Proteomics. tissue-based map of the human proteome. Science.

[12] Talbi S (2006). Molecular phenotyping of human endometrium distinguishes menstrual cycle phases and underlying biological processes in normo-ovulatory women. Endocrinology.

[13] Fu BQ (2017). Natural killer cells promote fetal development through the secretion of growth-promoting factors. Immunity.

[14] Cousins FL, Kirkwood PM, Saunders PTK, Gibson DA (2016). Evidence for a dynamic role for mononuclear phagocytes during endometrial repair and remodelling. Sci. Rep..

[15] Wang W (2020). Single-cell transcriptomic atlas of the human endometrium during the menstrual cycle. Nat. Med..

[16] Vento-Tormo R (2018). Single-cell reconstruction of the early maternal-fetal interface in humans. Nature.

[17] Bilyk O, Coatham M, Jewer M, Postovit LM (2017). Epithelial-to-mesenchymal transition in the female reproductive tract: from normal functioning to disease pathology. Front Oncol..

[18] Yue K (2015). Synthesis, properties, and biomedical applications of gelatin methacryloyl (GelMA) hydrogels. Biomaterials.

[19] Fassnacht M (2015). Linsitinib (OSI-906) versus placebo for patients with locally advanced or metastatic adrenocortical carcinoma: a double-blind, randomised, phase 3 study. Lancet Oncol..

[20] Diaz-Gimeno P, Ruiz-Alonso M, Blesa D, Simon C (2014). Transcriptomics of the human endometrium. Int J. Dev. Biol..

[21] Evans GE (2014). In the secretory endometria of women, luminal epithelia exhibit gene and protein expressions that differ from those of glandular epithelia. Fertil. Steril..

[22] Proserpio V, Lonnberg T (2016). Single-cell technologies are revolutionizing the approach to rare cells. Immunol. Cell Biol..

[23] Wu B (2017). Reconstructing lineage hierarchies of mouse uterus epithelial development using single-cell analysis. Stem Cell Rep..

[24] Krjutskov K (2016). Single-cell transcriptome analysis of endometrial tissue. Hum. Reprod..

[25] Garcia-Alonso L (2021). Mapping the temporal and spatial dynamics of the human endometrium in vivo and in vitro. Nat. Genet..

[26] Koh W (2019). Single cell transcriptomes derived from human cervical and uterine tissue during pregnancy. Adv. Biosyst..

[27] Fitzgerald HC, Dhakal P, Behura SK, Schust DJ, Spencer TE (2019). Self-renewing endometrial epithelial organoids of the human uterus. Proc. Natl Acad. Sci. USA.

[28] Lv Q, Wang L, Luo X, Chen X (2021). Adult stem cells in endometrial regeneration: Molecular insights and clinical applications. Mol. Reprod. Dev..

[29] Plikus MV (2021). Fibroblasts: Origins, definitions, and functions in health and disease. Cell.

[30] Ogo Y (2014). IGF-1 gene expression is differentially regulated by estrogen receptors alpha and beta in mouse endometrial stromal cells and ovarian granulosa cells. J. Reprod. Dev..

[31] Slavin BR (2021). Insulin-like growth factor-1: a promising therapeutic target for peripheral nerve injury. Front Bioeng. Biotechnol..

[32] Hossain MA (2021). IGF-1 facilitates cartilage reconstruction by regulating PI3K/AKT, MAPK, and NF-kB signaling in rabbit osteoarthritis. J. Inflamm. Res..

[33] Wang L (2018). Transplant of insulin-like growth factor-1 expressing bone marrow stem cells improves functional regeneration of injured rat uterus by NF-kappaB pathway. J. Cell Mol. Med.

[34] Turco MY (2017). Long-term, hormone-responsive organoid cultures of human endometrium in a chemically defined medium. Nat. Cell Biol..

[35] Ramilowski JA (2016). A draft network of ligand-receptor-mediated multicellular signalling in human. Nat. Commun..

[36] Diaz-Mejia, J. J. et al. Evaluation of methods to assign cell type labels to cell clusters from single-cell RNA-sequencing data. *F1000Res* **8**, ISCB Comm J-296 (2019). 10.12688/f1000research.18490.1 PMC6720041 31508207

[37] Hong Y (2019). A strongly adhesive hemostatic hydrogel for the repair of arterial and heart bleeds. Nat. Commun..

