# Supplementary material for: SFRP4+ stromal cell subpopulation with IGF1 signaling in human endometrial regeneration
Source: Cell Discov. 2022 Sep 27;8:95. doi: 10.1038/s41421-022-00438-7 (PMC9512788; doi:10.1038/s41421-022-00438-7)
Supplement: Supplementary file 1 — Supplementary Figure S1-S11, Table S1 [file 41421_2022_438_MOESM1_ESM.pdf]

Supplementary Figures:

Supplementary Figure S1. Quality control of single cell profiling and unbiased clustering. The number of counts, feature and mitochondrial counts of each cell from the proliferative (NP) and secretory (NS) phase uterus samples before **(a & c)** and after **(b & d)** the data were filtered. Then we selected most variable expressed genes by selecting the most variable principal component (PC) **(e)** to perform graph-based clustering of the combined dataset. **(f)**. The potential doublet of the single cell data was detected using DoubleFinder.

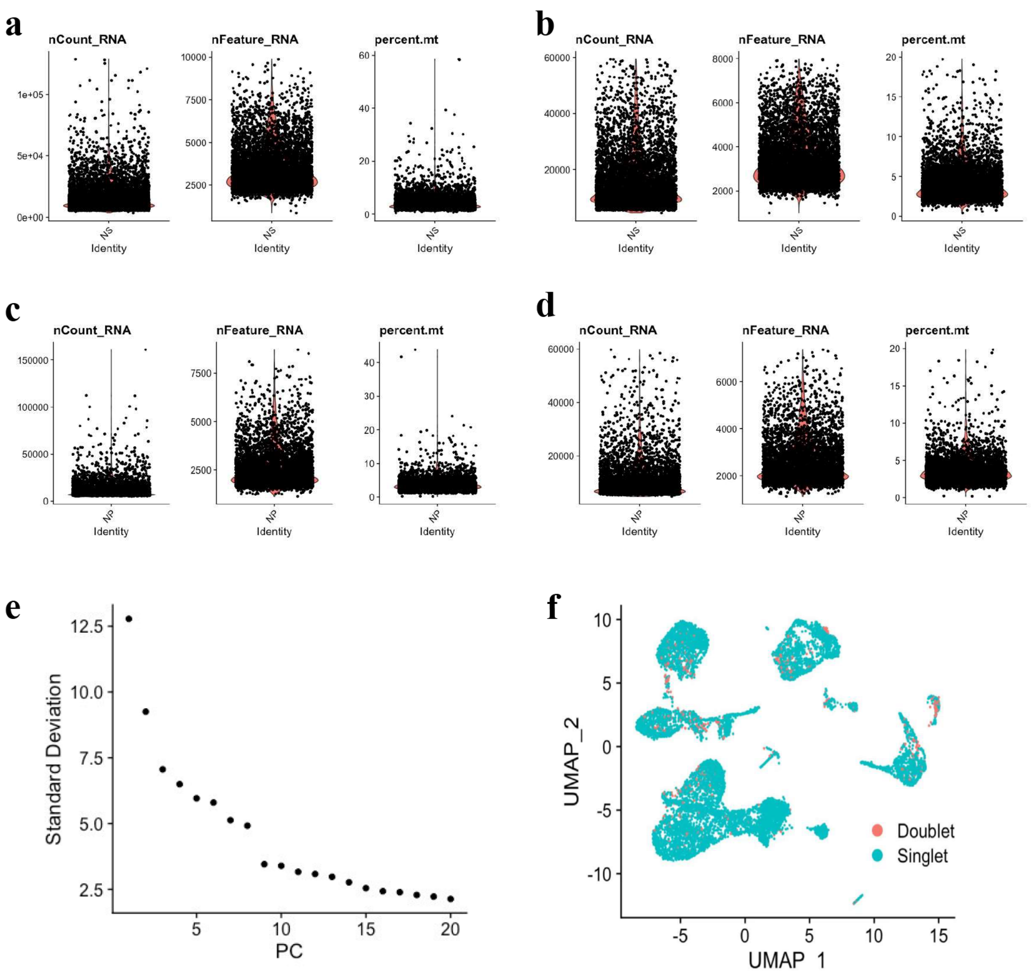

Supplementary Figure S2. Heterogeneity of uterine cell populations. (a) (left) UMAP plot of 5 uterus epithelial cell sub-populations using Seurat. Heatmap shows specifically expressed gene signature of 5 uterus epithelial cell sub-populations. (right) Gene ontology (GO) analysis of the specifically expressed gene signature of each sub-population from epithelial cells. **(b)** (left) UMAP plot of 2 uterus endothelia cell sub-populations using Seurat. Heatmap shows specifically expressed gene signature of each sub-population from endothelia cells. (right) GO analysis of the specifically expressed gene signature of each sub-population from endothelia cells. **(c)** (left) UMAP plot of 4 uterus vascular smooth muscle cell sub-populations using Seurat. Heatmap shows specifically expressed gene signature of each vascular smooth muscle cell sub-population. (right) GO analysis of the specifically expressed gene signature of each vascular smooth muscle cell. **(d)** (left) UMAP plot of uterus immune cell sub-populations using Seurat. Heatmap shows specifically expressed gene signature of sub-populations from immune cells. (right) GO analysis of the specifically expressed gene signature of the immune cell sub-populations. **(e)** (left) UMAP plot of uterus myometrial smooth muscle cell sub-populations using Seurat. Heatmap shows specifically expressed gene signature of 3 myometrial smooth muscle cell sub-populations. (right) GO analysis of the specifically expressed gene signature of the three myometrial smooth muscle cell sub-populations.

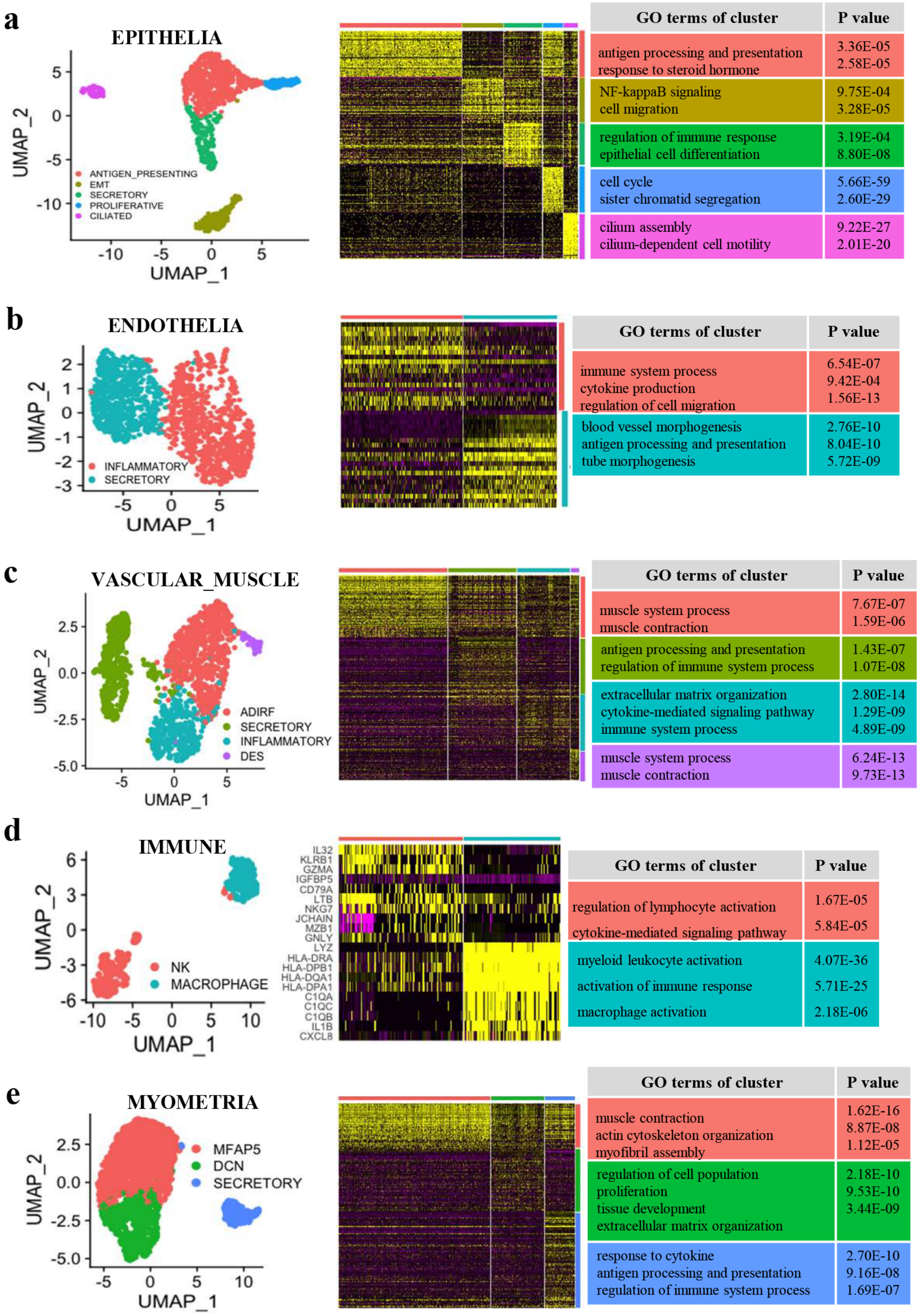
a
EPITHELIA
UMAP_2
UMAP_1
ANTIGEN_PRESENTING
EMT
SECRETORY
PROLIFERATIVE
CILIATED
GO terms of cluster
P value
antigen processing and presentation
response to steroid hormone
3.36E-05
2.58E-05
NF-kappaB signaling
cell migration
9.75E-04
3.28E-05
regulation of immune response
epithelial cell differentiation
3.19E-04
8.80E-08
cell cycle
sister chromatid segregation
5.66E-59
2.60E-29
cilium assembly
cilium-dependent cell motility
9.22E-27
2.01E-20
b
ENDOTHELIA
UMAP_2
UMAP_1
INFLAMMATORY
SECRETORY
GO terms of cluster
P value
immune system process
cytokine production
regulation of cell migration
6.54E-07
9.42E-04
1.56E-13
blood vessel morphogenesis
antigen processing and presentation
tube morphogenesis
2.76E-10
8.04E-10
5.72E-09
c
VASCULAR_MUSCLE
UMAP_2
UMAP_1
ADIRF
SECRETORY
INFLAMMATORY
DES
GO terms of cluster
P value
muscle system process
muscle contraction
7.67E-07
1.59E-06
antigen processing and presentation
regulation of immune system process
1.43E-07
1.07E-08
extracellular matrix organization
cytokine-mediated signaling pathway
immune system process
2.80E-14
1.29E-09
4.89E-09
muscle system process
muscle contraction
6.24E-13
9.73E-13
d
IMMUNE
UMAP_2
UMAP_1
NK
MACROPHAGE
IL32
KLRB1
GZMA
IGFBP5
CD79A
LTB
NKG7
JCHAIN
MZB1
GNLY
LYZ
HLA-DRA
HLA-DPB1
HLA-DQA1
HLA-DPA1
C1QA
C1QC
C1QB
IL1B
CXCL8
GO terms of cluster
P value
regulation of lymphocyte activation
cytokine-mediated signaling pathway
1.67E-05
5.84E-05
myeloid leukocyte activation
activation of immune response
macrophage activation
4.07E-36
5.71E-25
2.18E-06
e
MYOMETRIA
UMAP_2
UMAP_1
MFAP5
DCN
SECRETORY
GO terms of cluster
P value
muscle contraction
actin cytoskeleton organization
myofibril assembly
1.62E-16
8.87E-08
1.12E-05
regulation of cell population
proliferation
tissue development
extracellular matrix organization
2.18E-10
9.53E-10
3.44E-09
response to cytokine
antigen processing and presentation
regulation of immune system process
2.70E-10
9.16E-08
1.69E-07

Supplementary Figure S3. Flowchart shows deconvolution analysis was conducted using marker genes of each subpopulation generated in our cell atlas as gene set, and calculate the relative proportional score of each subpopulation in endometria during the menstrual cycle from another transcriptional dataset of the human endometria during the menstrual cycle (GSE4888).

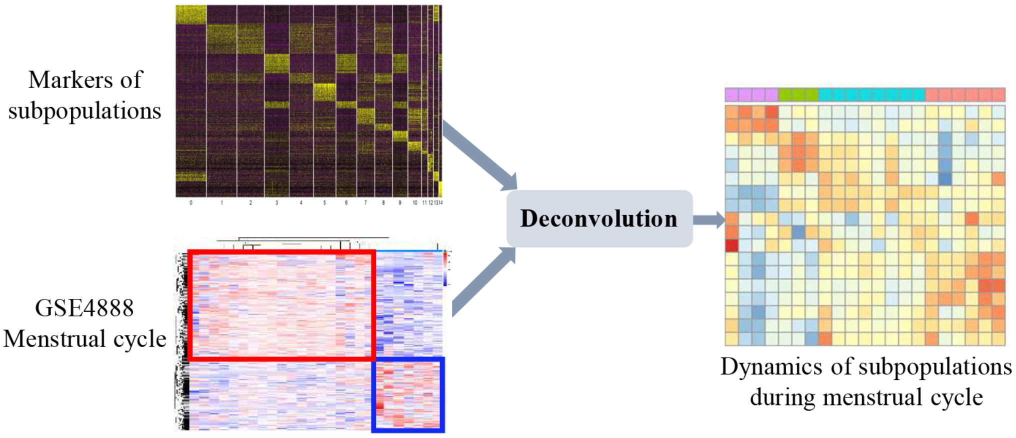

Supplementary Figure S4. Human endometrium single cell sequencing verified SFRP4+ stroma as proliferative phase specific endometrial cell populations. (a) UMAP plot of 5 uterus stroma cell sub-populations using Seurat. **(b)** Featureplot depicted specific markers for each stroma sub-population (*SCGB1D2* for secretory stroma cells; *SFRP4* for SFRP4+ stroma cells; *DCN* for two DCN+ stroma cells; *ACTA2* for myofibroblast cells). **(c)** Different proportion of stromal subsets in different phases during menstrual cycle.

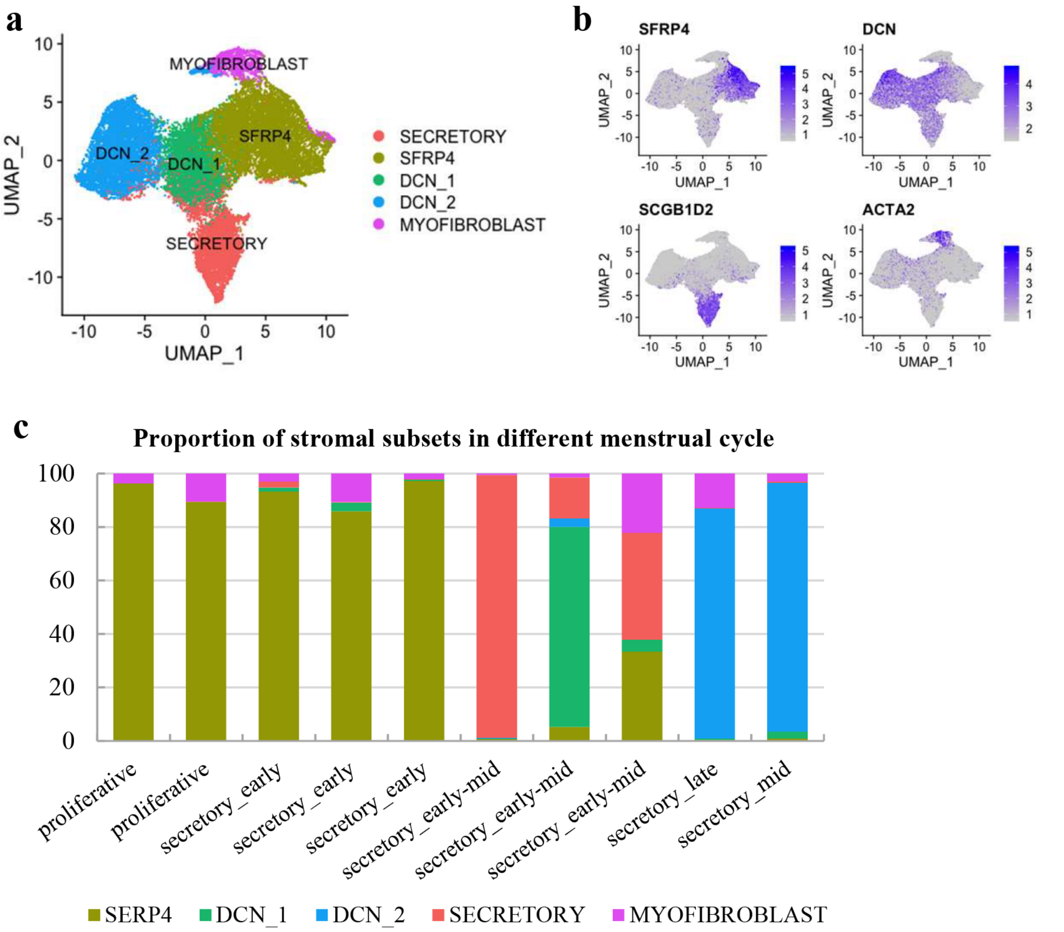

Supplementary Figure S5. Spatial distribution of SFRP4 + stromal cells. Immunohistochemistry staining from the HPA showed the spatial distribution of SFRP4 + stromal cells in the functional endometrial layer close to the uterine cavity **(a)**, and basal endometrial layer close to the myometrium **(b)**, SFRP4 (https://www.proteinatlas.org/ENSG00000106483-SFRP4/tissue/endometrium#), Scale bar, 100 μm.

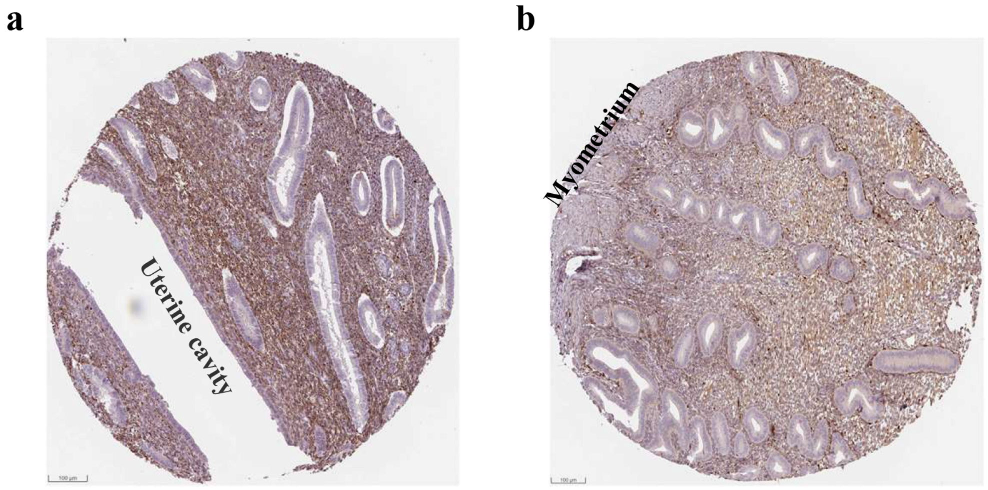

Supplementary Figure S6. Connectivity analysis reveal distinct microenvironment signals in secretory phase. (a) Heatmap shows the total numbers of receptor-ligand interactions from any of the two sub-populations from the microenvironment of full-thickness human uterus in secretory phase. **(b)** Heatmap shows expression of ligands uniquely expressed in mid- or late-secretory phase of the human endometria derived from another transcriptional dataset of the human endometria during the menstrual cycle (GSE4888). **(c)** Clustering of the interactions of ligands (uniquely expressed in secretory endometria) from each sub-population of the secretory endometria to receptors from the EMT epithelial cell sub-population.

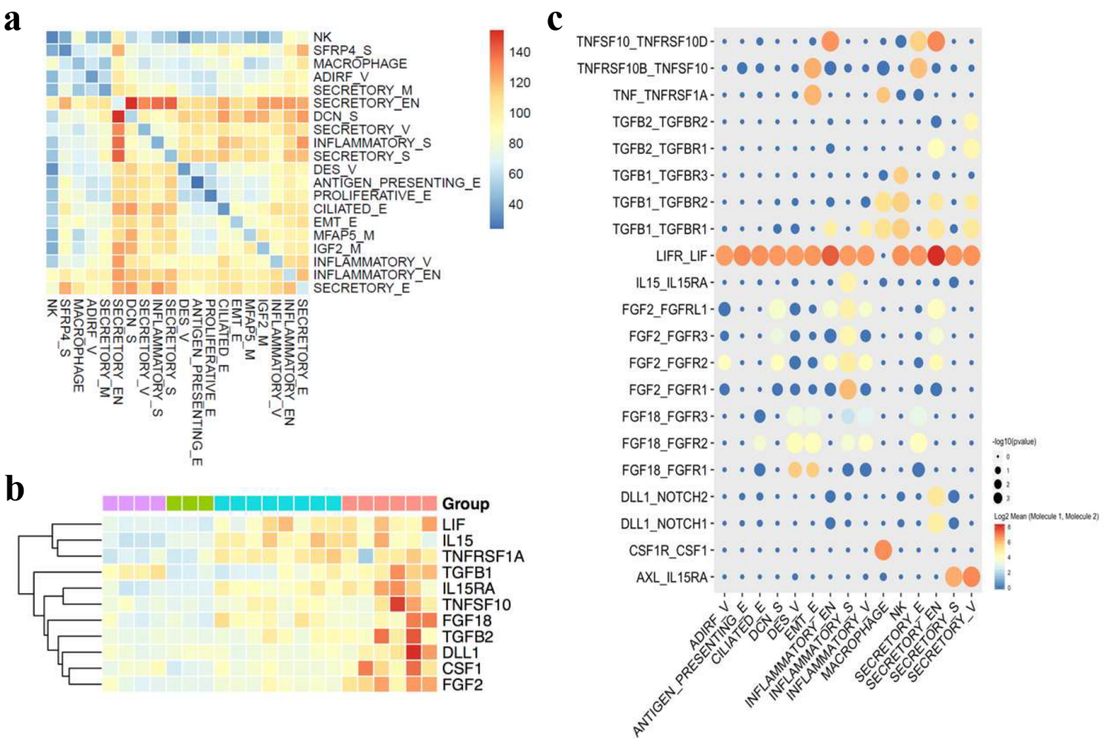

Supplementary Figure S7. IGF1 promotes the proliferation and migration of endometrial epithelial cells. **(a)** organoids culturing of endometrial epithelia cells with or without 100ng/ml IGF1 added in combination to generic organoid medium (ENR), scale bar=200μm. **(b)** Quantification of endometrial epithelial organoids in different groups, ***, p value<0.001; **(c)** radius of endometrial epithelial organoids in different groups, ***, p value<0.001. **(d-e)** Trajectory of endometrial epithelial cells monitored by CV1000 Live cell Imaging System. **(f)** velocity of endometrial epithelial cells, ***, p value<0.001.

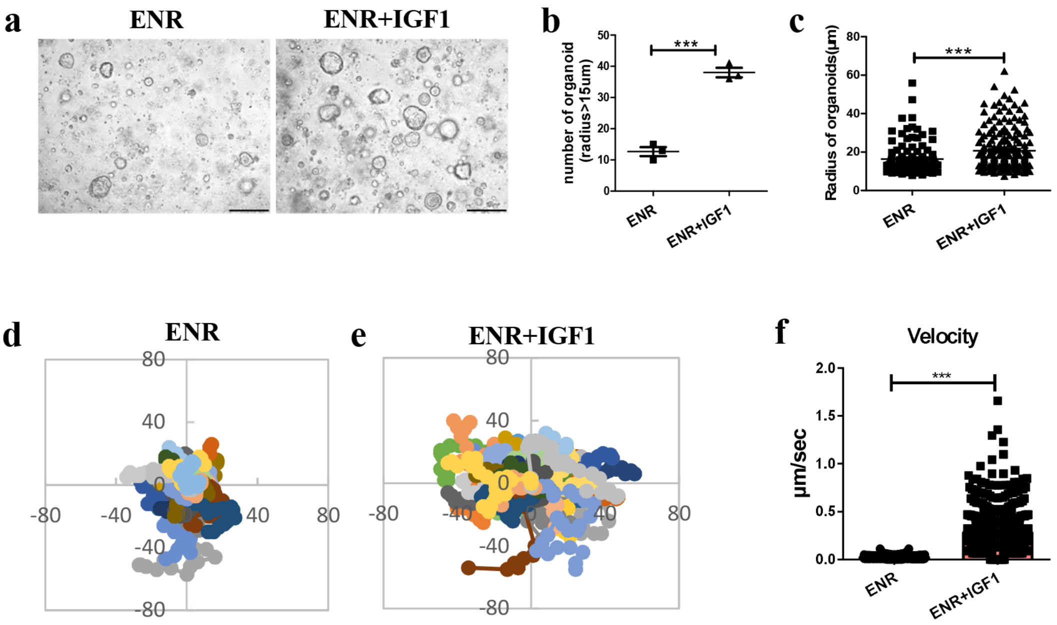

Supplementary Figure S8. The impact of IGF1 inhibition/knockdown on stromal cell proliferation and methods for quantifying organoid number and diameter. (a) Three different siRNA were designed according to different regions of the human *IGF1* mRNA (IGF1-si-1, IGF1-si-2, IGF1-si-3) to knock down *IGF1* in the SFRP4$^+$ stromal cells, 24 hours after transfection, QPCR was conducted to validated the efficiency of each siRNA, the results showed that both IGF1-si-1 and IGF1-si-3 could significantly knock down the expression of *IGF1* in the SFRP4$^+$ stromal cells, with the IGF1-si-3 showed the best performance, so we chose IGF1-si-3 to do the rest of the experiments. n=3, *, p value<0.05. **(b)** The impact of IGF1 siRNA knockdown on stromal cell proliferation. **(c)** The impact of IGF1 signaling inhibition using inhibiotrs on stromal cell proliferation. n=6, *p value<0.05, **p value<0.01, ***p value<0.001. **(d)** The endometrial epithelial organoid spheres in the pictures are automatically recognized and selected with the count and measure objects function plug-in in Image-Pro Plus 6.0 (the red logo is the endometrial epithelial organoid automatically recognized by the software), and the software calculates the number of epithelial organoids and the diameter of each epithelial organoid ball at the same time for further statistical analysis.

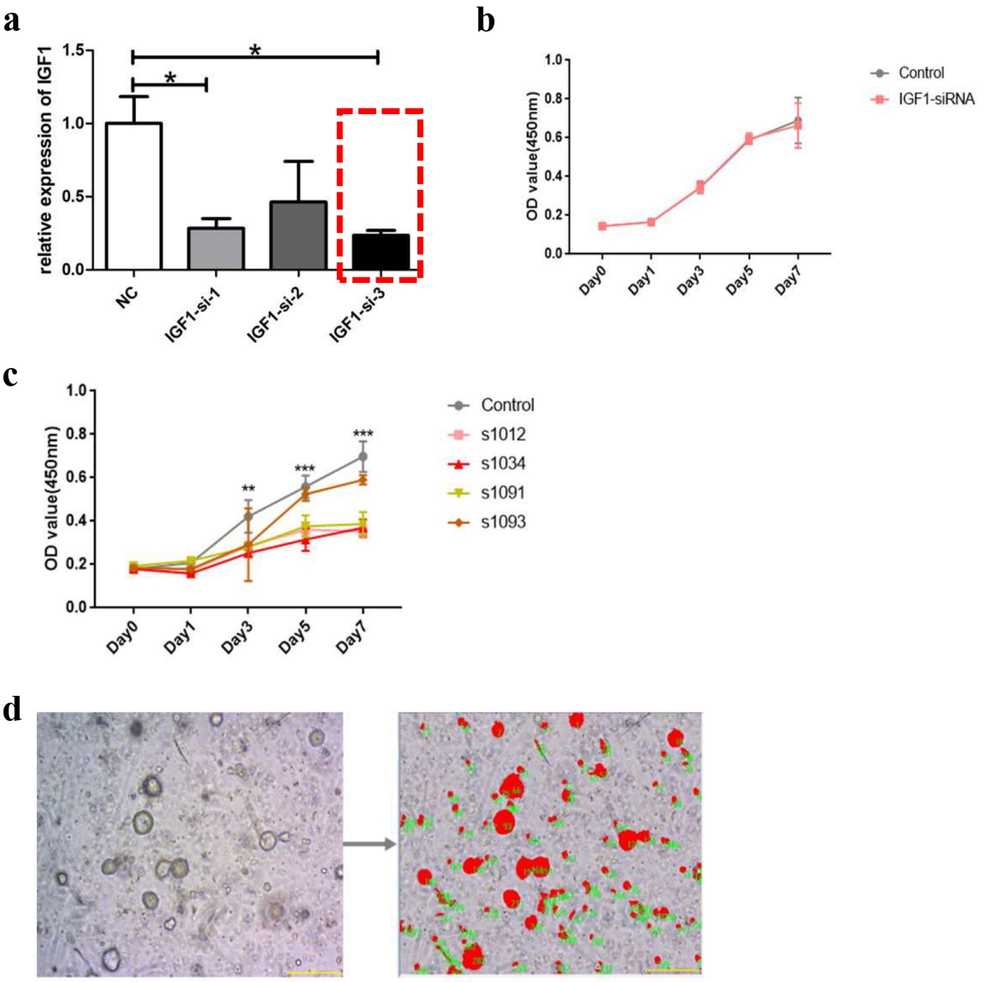
a
relative expression of IGF1
1.5
1.0
0.5
0.0
*
*
NC
IGF1-si-1
IGF1-si-2
IGF1-si-3
b
OD value(450nm)
1.0
0.8
0.6
0.4
0.2
0.0
Day0
Day1
Day3
Day5
Day7
Control
IGF1-siRNA
c
OD value(450nm)
1.0
0.8
0.6
0.4
0.2
0.0
**
***
***
Day0
Day1
Day3
Day5
Day7
Control
s1012
s1034
s1091
s1093
d

Supplementary Figure S9. The endometrial injury model. The endometrial injury model with endometrial layers torn off with the smooth muscle layer remained intact was performed as follows after animal anesthetized: a midline incision in the abdomen was made, and the uterus was exposed. In the injury alone group, A 1cm longitudinal incision was made on the opposite side of the mesometrium with the endometrial layer exposed. Then the endometrial layers with the size of 1cm in length and 0.5cm in width were torn off with the smooth muscle layer remained intact.

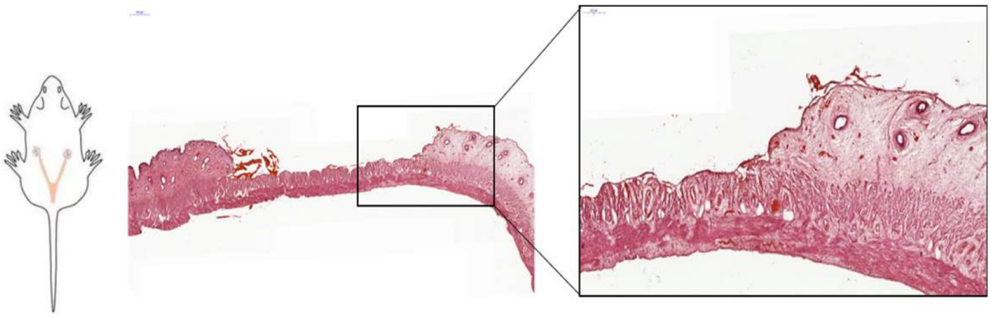

Supplementary Figure S10. Characterization of GelMA. **(a)** gelatinization after UV irradiation for 15s; **(b)** 1H-NMR spectra of gelatin and GelMA. **(c)** SEM (scanning electron microscope) of GelMA; **(d)** cell viability of cells cultured in GelMA hydrogel, in which the red was dead cell, green was living cell (2D:2 dimension image, 3D:3 dimension imaging); **(e)** The percentage of cell viability at different time points (**, P value<0.01, ns, no significance). **(f)** CCK8 testing of cell proliferation when cultured in GelMA hydrogel extract.

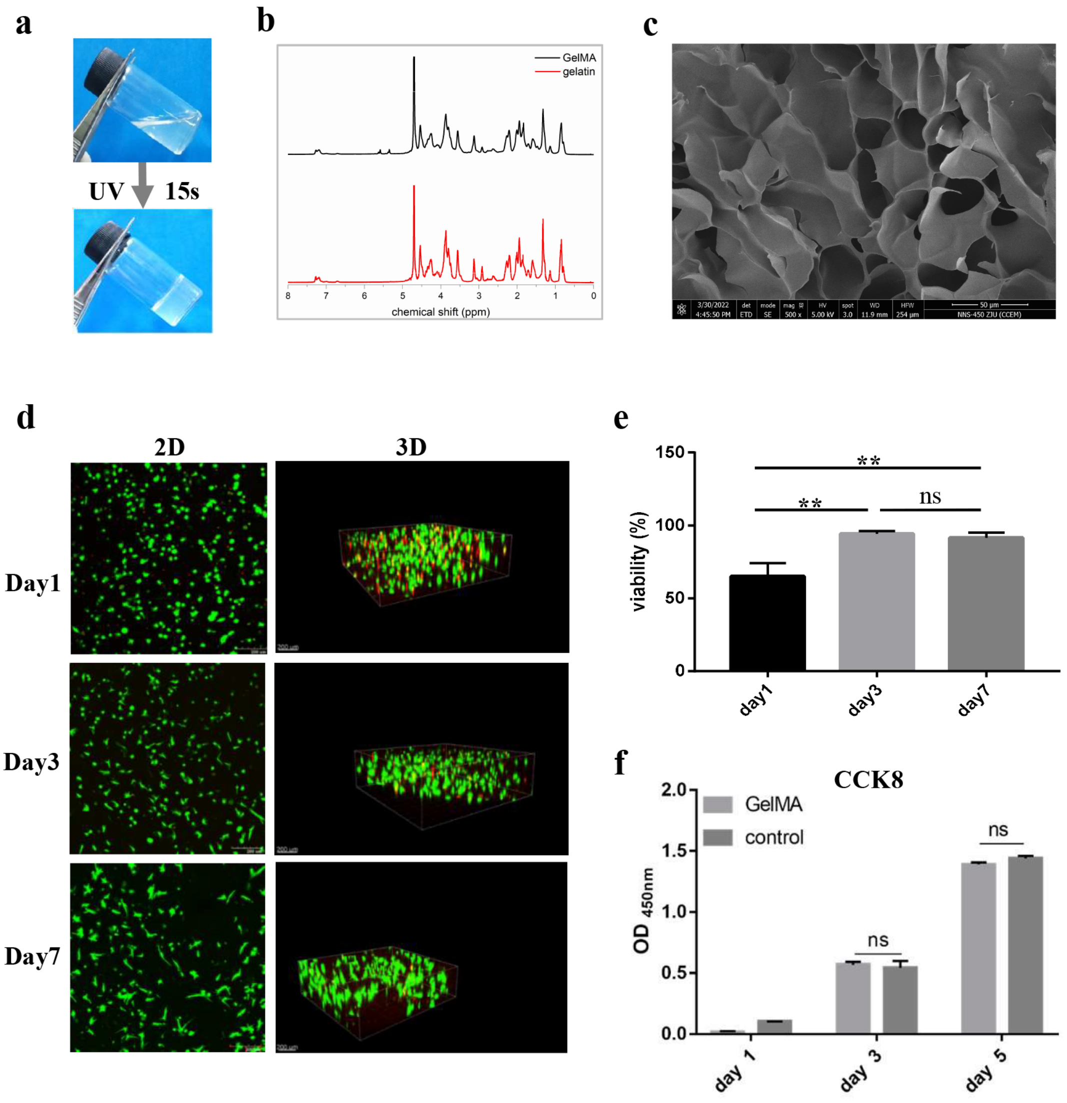

Supplementary Figure S11. Marker expression in the subpopulations of in our study. **(a)** the expression of markers of the four epithelial clusters of the Nat Genet 2021 paper were mapped in our epithelial data. **(b)** the expression of markers of the three fibroblast/stromal clusters in the Nat Genet 2021 paper were mapped in our stromal cells data. **(c)** SUSD2 expression was highly enriched in our vascular cell population (ADIRF+ vascular cells). **(d)** There's no positive SUSD2 expression in any of the stroma cells in our data.

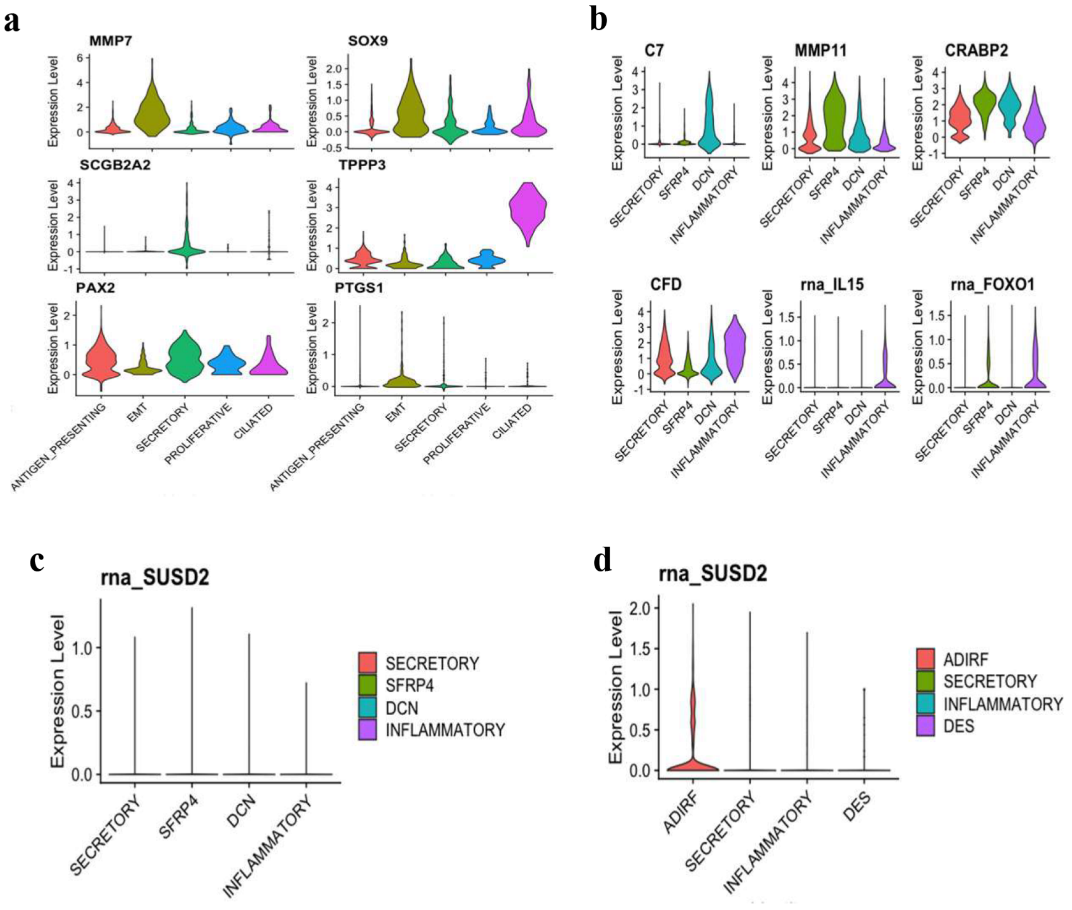

Supplementary Tables:

Supplement table 1. Human full-thickness uterus specimen collection

Samples	Age	Menstrual phase	Menstrual cycle (days)
**Sample1***	45	Proliferative	25
**Sample2*#**	46	Proliferative	28-30
**Sample3**	51	Proliferative	27
Sample4*	44	Proliferative	25
Sample5*	49	Proliferative	28
**Sample6***	49	Secretory	30
**Sample7**	44	Secretory	25
**Sample8**	44	Secretory	30
**Sample9***	44	Secretory	25-26
Sample10*	46	Secretory	28
Sample11*	46	Secretory	30
Sample12#	46	Proliferative	20-30

The samples marked in bold were used for single cell analysis. The samples marked with an asterisk were used for immunofluorescence analysis. The samples marked with # indicated cell isolations for organoid experiments.

Supplementary Table S2. Specific markers of each of the main cluster of the human uterus

Supplementary Table S3. Gene ontology of the specific markers of each of the main cluster of the human uterus

Supplementary Table S4. Specific markers of each of the epithelial sub-population of the human uterus

Supplementary Table S5. Gene ontology of the specific markers of each of the epithelial sub- population of the human uterus

Supplementary Table S6. Specific markers of each of the stromal sub-population of the human uterus

Supplementary Table S7. Gene ontology of the specific markers of each of the stromal sub- population of the human uterus

Supplementary Table S8. Specific markers of each of the endothelial sub-population of the human uterus

Supplementary Table S9. Gene ontology of the specific markers of each of the endothelial sub- population of the human uterus

Supplementary Table S10. Specific markers of each of the vascular muscle sub-population of the human uterus

Supplementary Table S11. Gene ontology of the specific markers of each of the vascular muscle sub-population of the human uterus

Supplementary Table S12. Specific markers of each of the immune cell sub-population of the human uterus

Supplementary Table S13. Gene ontology of the specific markers of each of the immune cell sub-population of the human uterus

Supplementary Table S14. Specific markers of each of the myometrial muscle cell sub-population of the human uterus

Supplementary Table S15. Gene ontology of the specific markers of each of the myometrial muscle cell sub-population of the human uterus

Supplementary Table S16. Significant connections of ligand-receptor pairs from cell sub- populations from the proliferative phase of the human uterus. This table provides the list of ligand-receptor interactions pairs between each two sub-populations from the ecosystem of the proliferative phase of the human uterus

Supplementary Table S17. Significant connections of ligand-receptor pairs from

cell sub- populations from the secretory phase of the human uterus. This table provides the list of ligand-receptor interactions pairs between each two sub-populations from the ecosystem of the secretory phase of the human uterus
